# Potential impacts of policies to reduce purchasing of ultra-processed foods in Mexico at different stages of the social transition: an agent-based modeling approach

**DOI:** 10.1017/S1368980021004833

**Published:** 2021-12-13

**Authors:** Brent A. Langellier, Ivana Stankov, Ross A. Hammond, Usama Bilal, Amy H. Auchincloss, Tonatiuh Barrientos-Gutierrez, Leticia de Oliveira Cardoso, Ana V. Diez Roux

**Affiliations:** 1 Department of Health Management and Policy, Dornsife School of Public Health, Drexel University, Philadelphia, Pennsylvania, USA; 2 Urban Health Collaborative, Dornsife School of Public Health, Drexel University, Philadelphia, Pennsylvania, USA; 3 Center on Social Dynamics & Policy, Brookings Institution, Washington, DC, USA and Public Health and Social Policy, Washington University in St. Louis, St. Louis, Missouri, USA; 4 Department of Epidemiology and Biostatistics, Dornsife School of Public Health, Drexel University, Philadelphia, Pennsylvania, USA; 5 Center for Population Health Research. National Institute of Public Health. Cuernavaca, México; 6 Oswaldo Cruz Foundation, Rio de Janeiro, Brazil; 7 UniSA Allied Health and Human Performance, University of South Australia, Adelaide, South Australia, Australia

**Keywords:** Food policy, diet, complex systems, social determinants of health, simulation

## Abstract

**Objectives:**

To develop a simulation framework for assessing how combinations of taxes, nutrition warning labels, and advertising levels could affect purchasing of ultra-processed foods (UPFs) in Latin American countries, and to understand whether policies reinforce or reduce pre-existing social disparities in UPF consumption.

**Design:**

We developed an agent-based simulation model using international evidence regarding the effect of price, nutrition warning labels, and advertising on UPF purchasing.

**Setting:**

We estimated policy effects in scenarios representing two stages of the “social transition” in UPF purchasing: 1) a pre-transition scenario, where UPF purchasing is higher among high-income households, similar to patterns in Mexico, 2) a post-transition scenario where UPF purchasing is highest among low-income households, similar to patterns in Chile.

**Participants:**

A population of 1,000 individual-agents with levels of age, income, educational attainment, and UPF purchasing similar to adult women in Mexico.

**Results:**

A 20% tax would decrease purchasing by 24% relative to baseline in both the pre- and post-transition scenarios, an effect that is similar in magnitude to that of a nutrition warning label policy. A 50% advertising increase or decrease had a comparatively small effect. Nutrition warning labels were most effective among those with higher levels of educational attainment. Labeling reduced inequities in the pre-transition scenario (i.e., highest UPF purchasing among the highest socioeconomic group), but widened inequities in the post-transition scenario.

**Conclusions:**

Effective policy levers are available to reduce UPF purchasing, but policymakers should anticipate that equity impacts will differ depending on existing social patterns in UPF purchasing.

## Introduction

Ultra-processed foods (UPFs) are an increasingly dominant part of the global food system,^(1)^ and their availability and consumption have increased in most countries and regions.^(2–5)^ UPFs are foods that have been developed via “fractioning of whole foods into substances, chemical modifications of these substances, assembly of unmodified and modified food substances, frequent use of cosmetic additives and sophisticated packaging.”^(6)^ Engineered to maximize profit margins, convenience, shelf stability, and palatability relative to unprocessed or minimally processed foods,^(7)^ they tend to have more added sugar, more saturated fat, more sodium, less fiber, less micronutrients and much higher energy density.^(8, 9)^ UPF consumption is positively associated with development of obesity among youth and adults.^(10, 11)^

In many Latin American countries, calories from UPFs contribute 20-30% or more to total energy intake.^(3, 5)^ Latin American countries have been at the forefront of using policy levers to address rising UPF consumption, including UPF taxes, front-of-package nutrition warning labels, and advertising restrictions.^(6, 8)^ In 2014, Mexico implemented an 8% tax on nonessential energy dense foods and a peso-per-liter (roughly equivalent to 10%) tax on sugar-sweetened beverages.^(12, 13)^ Purchasing of nonessential energy-dense foods fell 7.4% two years after implementation of the tax and purchasing of taxed beverages fell 9.7%.^(12, 13)^ Also in 2014, Chile increased an existing beverage tax from 13% to 18% for beverages high in sugar and reduced the tax rate from 13% to 10% for beverages low in sugar.^(14)^ As part of a front-of-package labeling and advertising law passed in 2016, Chile was the first country in the world to mandate front-of-package nutrition labels on energy dense foods, with other countries (including Peru and Uruguay) subsequently adopting their own labeling policies.^(15, 16)^ Two years after implementation of the Chilean law, purchasing of beverages high in added sugar decreased by 23.7%.^(15)^ Several countries in the region have also passed policies to address high levels of UPF advertising.^(17–19)^ For example, 14% of advertisements on the major “free to air” television channels in Brazil are food related, 91% of which are for UPF products.^(17)^

Evaluations of these vanguard policies in Latin America suggest that policy levers can meaningfully reduce UPF consumption at the population level. A remaining question is how policies can be used without creating or exacerbating existing inequities in UPF consumption. For example, evaluation data from Chile suggest that the labeling law had larger effects among individuals with high educational attainment who had lower consumption levels even prior to the tax.^(15)^ In contrast, the strongest impacts from the taxes implemented in Mexico were among households in the lower socioeconomic strata.^(20)^

The equity implications of the UPF reduction policies a country implements may depend on existing social patterns in UPF consumption, which vary between countries. UPF consumption is inversely associated with socioeconomic status in higher-income countries, but the reverse is true in lower- and middle-income countries.^(21)^ Baker and colleagues (2020) suggest that a “social transition” takes place as a country’s income distribution shifts upwards.^(21)^ In the first stage, the highest levels of UPF consumption are among individuals in the highest socioeconomic strata. As the income distribution shifts upwards, however, the highest levels of UPF consumption transition to those in the lower socioeconomic groups. Different Latin American countries may be in each stage. For example, higher-income households purchase more UPF than lower-income households in Mexico,^(3)^ but the reverse is true in Chile.^(15)^

Like many health behaviors, dietary choices are socially determined based on community-level social norms and social influence between peers, family members, and other close social contacts.^(22, 23)^ Policies to reduce UPF consumption will need to reverse secular trends that have made high levels of UPF consumption normative in most Latin American countries. Social influence on dietary choices likely contributes to the production and persistence of social inequities in UPF purchasing, particularly given international research suggesting a high degree of social homophily – meaning that strong social ties are most commonly formed between individuals with similar social characteristics, including age, educational attainment and income.^(24–26)^ In combination, social homophily and social influence are key mechanisms that contribute to the production and persistence of inequities between groups; these inequities can either be reduced or exacerbated by policies that have differential effects across social groups.^(15, 20)^

In this study, we report the results of an agent-based model (ABM) developed to understand the relative effectiveness of varying combinations of UPF tax, labeling, and advertising policies on UPF purchasing. We examine policy effects among a population of agents with social characteristics (age, income, educational attainment) that loosely represent female food purchasers in Mexican households. We consider policy effects in two broad scenarios: the first represents countries in which UPF purchasing is highest among high-income households (i.e., a pre-social transition in UPF purchasing similar to patterns observed in Mexico).^(21)^ In the second scenario, UPF purchasing is highest among low-income households (i.e., a post-social transition scenario that represents a plausible future scenario for Mexico and is qualitatively similar to patterns observed in Chile and in high-income countries). For each policy combination, we report the population-level effects, as well as stratified effects by income and educational attainment that are helpful for understanding how policies either reduce or exacerbate differences in UPF purchasing between socioeconomic strata.

## Methods

### Agent population and properties

We coded the model in NetLogo.^(27)^ Full details regarding model design, data sources, and effect parameters are in the model sketch in Appendix 2. We simulated weekly UPF purchasing, measured in kilocalories (kcal) purchased per week, among a virtual population of 1,000 individual-agents with levels of age, income, and educational attainment similar to those of adult women in Mexico. We selected weekly UPF purchasing in kcal because they are a widely used measure of energy intake, an important input to energy balance and weight change,^(28)^ and because they can be calculated across different types of UPF items (e.g., sweetened beverages, candy). We limited the population to adult women for three reasons: 1) women typically are the primary food purchasers in their household,^(29, 30)^ 2) adults and children have different food purchasing and consumption patterns, so focusing on adult women simplifies the parameters (e.g., distribution of UPF purchasing) and behavior rules in the model (e.g., social signal),^(3, 4)^ and 3) research on social homophily suggests that close social ties are most common among those of the same gender – including multiple genders in the model would unnecessarily complicate the social network formation and social signal, described below.^(24)^

Upon initialization of the model, each agent was assigned three characteristics that are important for food behaviors and social network formation: age group (young, middle-aged, older), income, and educational attainment. The age categories are intended to make a qualitative distinction between women in different life stages. We assigned one-quarter of agents to the younger and older age categories and 50% to the middle-aged category. Based on data from the 2016 Mexican Survey of Household Income and Spending (ENIGH), the categories generally correspond to women in the following age ranges: 1) 20 to 30, 2) 31 to 50, 3) >50. We used data from the 2016 ENIGH to inform the initial distributions of household income and educational attainment. Specifically, 27% of agents were assigned to the high education category, representing at least a high school education, and 73% to the low education category, representing less than high school. We assigned each agent a continuous household income drawn from separate log-normal distributions for those with low education (mean = 889 pesos per week, standard deviation = 911) and high education (mean = 2044, standard deviation = 2225). We then assigned agents with income above a threshold value (1890 pesos per week) to a high-income category and those below to the low-income category.^(31)^ We assigned both continuous and categorical income because the former is used in calculating relative UPF prices and the latter in social network formation. Agent properties and the data sources used to inform their distributions are summarized in **Table A-1** in Appendix 2.

### Baseline Scenarios: UPF purchasing

We examined policy effects under two baseline scenarios that represent pre- versus post-stages in the social transition in UPF purchasing.^(21)^ For both scenarios, we used data from Marrón-Ponce et al. (2019) to inform the initial distribution of UPF purchasing.^(32)^ Specifically, we calibrated the mean values among low- and high-income agents to reproduce the mean weekly purchasing among all household reported by Marrón-Ponce, which was 3,033 kcal per week. We calibrated the values for each income group because they were not reported by Marrón-Ponce or elsewhere. In the pre-transition scenario, the calibrated values at which we set mean UPF purchasing was 3,446 kcal per week for high-income agents and 2,966 kcal per week for low-income agents. This scenario is similar to existing patterns in UPF purchasing in Mexico, as reported by Marrón-Ponce. In the post-transition scenario, we set the mean calibrated UPF purchasing at 2,620 kcal per week for high-income agents and at 3,100 kcal per week for low-income agents. This is a counterfactual scenario for pre-transition countries, including Mexico, but is generally similar to patterns in post-transition countries, such as Chile.^(15)^

### Social network

Agents in the model were embedded in a small world network (with average node degree of 5.47), where the connections between agents represented close friendships between women in different households. Consistent with *in vivo* studies of social homophily,^(24–26, 33)^ agents were more likely to be connected to other agents with similar age, income, and educational attainment levels. Agents had at least three, and a maximum of 50 social connections. As shown in Table 1, we set the social network parameters to reproduce network characteristics similar to that reported in Chen (2019).

### Updates to UPF purchasing

Every time step, each agent in the model made a series of adjustments to her UPF purchasing level. The first adjustment represented the effects of a social signal and social norms.^(34, 35)^ The social signal can be thought of as representing processes of social learning and peer influence that occur between friends and family members.^(34)^ Social norms can be thought of as individuals’ desire to adhere to group-level norms among people with similar levels of age, income, and educational attainment.

For each adjustment, the agent calculated whether her own level of weekly UPF purchasing differed from the average among her social network by more than 50kcal/wk. The 50 kcal threshold represents uncertainty in people’s knowledge of the true levels of UPF purchasing of their friends. If the difference was less than the threshold the agent made no adjustment. If the difference was greater than the threshold, she shifted towards the social signal by a fractional amount (i.e., 10% of the difference). The same process was repeated for social norms, but each agent compared her purchasing to the average purchasing of all agents with the same levels of age (i.e., younger, middle aged, older), income (i.e., lower, higher) and education status (i.e., lower, higher).

The second type of update that agents made was in response to UPF policy changes. We used external data to inform the magnitude of these updates. The model calculated UPF purchasing in response to price changes based on the own-price elasticity of UPF. We set the own-price elasticity of UPF to -1.2 (i.e., a 1% increase in the price of UPF could be expected to produce a 1.2% decline in purchasing) based on studies of price elasticities of sugar-sweetened beverages in several Latin American countries, all of which range from -1.0 to -1.4.^(36–39)^ Notably, this range of values is within the range of effects observed at two years post-implementation of the UPF taxes implemented in Mexico (i.e., a 10-12% reduction at two years post-implementation of the ~10% tax).^(13, 20, 40)^ Based on the main evaluation study of the Chilean labeling policy,^(15)^ we set agents’ sensitivity to labeling (i.e., the difference in purchasing caused by a switch from no label to a front-of-package label) to -22% for low-education agents and - 29% for high-education agents. We set each agent’s sensitivity to advertising (i.e., the advertising elasticity) to 0.113 based on Hu, Lodish, and Krieger (2007).^(41)^ This means that a 1% increase in advertising translates to a 0.113% increase in purchasing. In simulation scenarios with multiple policy interventions, the change in purchasing from each of the policies is summed.

### Calibration

Calibration is an iterative process of making adjustments to parameter values such that model outcomes align with specified values, or calibration targets. We used calibration experiments to set the values of four unknown parameters: 1) resistance to conforming to the social signal and social norms, 2) the relative importance of social similarity (e.g., same age group) in constructing the social network, 3) the baseline level of UPF purchasing in low-income agents, and 4) the baseline level of UPF purchasing in high-income agents. The first two parameters were the same in the pre- and post-social transition scenarios, and only the third and fourth differed. The calibration target we used was that the average, equilibrium state weekly household UPF purchasing at the population-level must be within 5 kCal of the calibration target of 3,033 kCal per week based on findings reported in Marrón-Ponce et al (2019).^(32)^ In the pre-social transition scenario, we required that low income households had lower weekly UPF consumption than high income households, and vice-versa for the post-social transition scenario. We also required that the calibrated parameters produce unique distributions of weekly household UPF purchasing by income and that these distributions remained unique (i.e., did not fully converge) as the model ran.

### Policy Counterfactuals

We used the ABM to examine how UPF purchasing would be affected by a UPF labeling policy and UPF taxes of 8% (actual junk food tax in Mexico), 20% (beverage tax level considered in Mexico but not passed) and 50% (counterfactual “high tax” scenario). Several countries in the Arabian peninsula have implemented taxes of 50% or more on sweetened beverages and excises taxes implemented in several local areas in the United States (e.g., 1.5, 1.75, and 2.0 cents per ounce in Philadelphia, Seattle, and Boulder, respectively) equate to over 50% for some products.^(42, 43)^ These examples suggest that, though taxes in this amount have not been implemented in Latin American countries, they are of a level that could plausibly be considered by policymakers at both the local or national level. We also examined how policy effects would be impacted by increases in UPF advertising of 25% and 50%, representing industry responses to labeling and tax policies, as well as similar decreases in UPF advertising, representing policy restrictions on advertising. For both the pre- and post-social transition scenarios, we ran iterations of the model with no policy, with each policy implemented alone, and with multiple policy combinations.

The model ran in discrete time, with each time step representing one week. We compared mean UPF purchasing in each scenario after 208 time steps (i.e., four years), not including a burn-in period of 100 time steps. The burn-in period allowed each agent to update her food purchasing until the model reached a stable state that aligned with population-level UPF purchasing trends (calibration target) reported by Marrón-Ponce et al (2019).^(32)^ We implemented policies one year into the simulation (i.e., after 52 time steps) and ran each scenario 200 times to account for random variation. This number of runs was determined through sensitivity analyses which suggested that only relatively small variations in weekly UPF purchasing were observed for simulations higher than 200 runs (see Appendix Figure 2-A).

## Results

**Figure 1:**
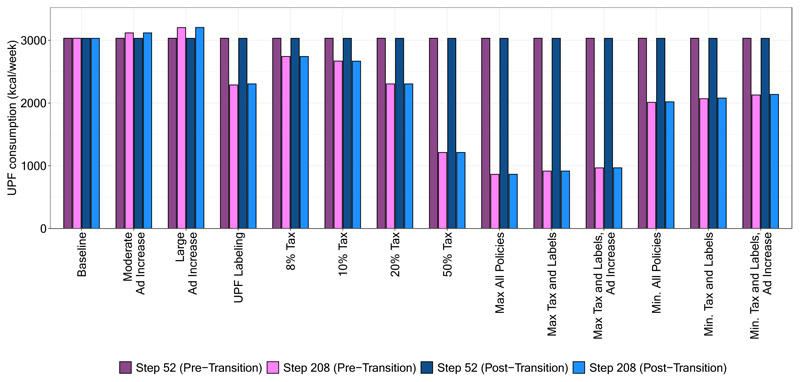
Mean weekly purchasing of ultra-processed foods in kCal/week at time steps 52 (just prior to policy implementation) and 208 (equivalent to 3 years post-implementation), by policy scenario and stage of the social transition in UPF purchasing

In Figure 1, we show mean UPF purchasing under different policy combinations at time steps 52 (just prior to policy implementation) and 208 (~3 years after implementation) in both the pre- and post-social transition scenarios. In both the post- and pre-social transition scenarios, mean UPF purchasing demonstrated good fit to the average weekly UPF purchasing (i.e., 3,033 kCal per week) estimated by Marrón-Ponce et al. (2019). Generally, the population-level effects of each policy were similar between the post- and pre-transition scenarios.

Among the policies implemented alone, the 50% tax produced the largest decrease in UPF purchasing, a decrease of about 60% relative to the baseline in the both the pre- and post-transition scenarios. The labeling policy and the 20% tax each had similar effects when implemented alone, decreasing UPF purchasing by about 24%. Notably, the effect of a 50% change in advertising was much smaller than the tax or labeling policies – for example, a 50% increase in advertising resulted in an increase in UPF purchasing by 6%. Among policies implemented in combination, the scenario that included a 50% tax, labeling, and a 50% reduction in advertising reduced UPF purchasing by 72%. This scenario assumes that the amount of advertising promoting UPF purchasing would be reduced, likely through a policy change that limited or taxed industry advertising. Without any change in advertising, the 50% tax and label policy still decreased UPF purchasing by 70%. Even if industry responded to the tax and labeling policies by *increasing* advertising levels by 50%, UPF purchasing would still be lower than the baseline scenario by 68%.

The 8% UPF tax, which resembles the junk food tax implemented in Mexico, decreased weekly UPF purchasing by about 10% relative to pre-implementation levels. If Mexico were to also implement a UPF labeling policy, the model estimates a reduction in UPF purchasing by about 31% (approximately 2,000 kcal per week). Even if industry responded by increasing UPF advertising by 25%, the reduction in UPF purchasing would be very similar (just under 30% or approximately 2,100 calories per week).

**Figure 2:**
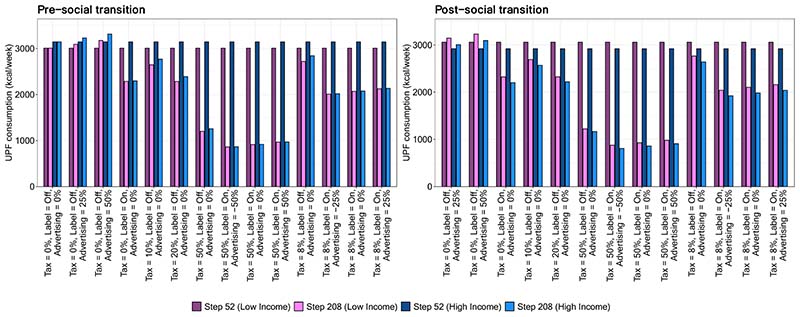
Mean weekly purchasing of ultra-processed foods in kCal/week by income strata, in a population in which UPF purchasing is greater in households with either higher income (pre-social transition, left panel) or lower income (post-social transition, left panel)

**Figure 3:**
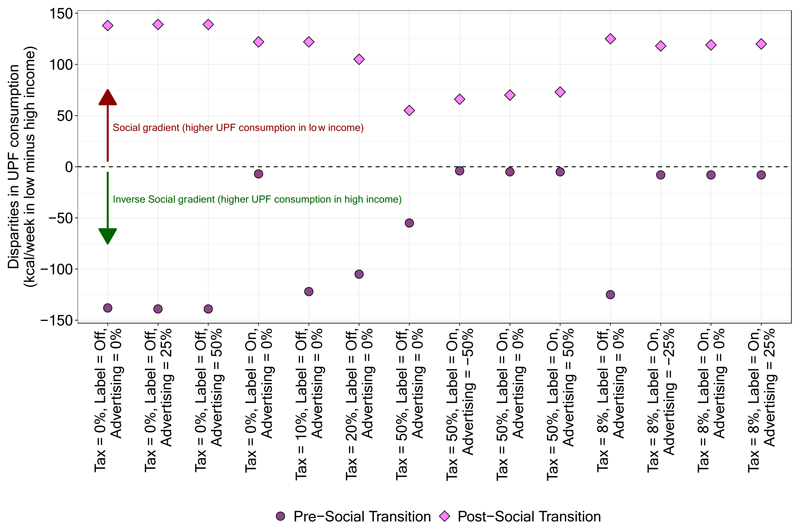
Difference in weekly purchasing of ultra-processed foods between the lower and higher income strata at time step 208, by stage of the social transition.

In Figure 2, we report estimated UPF purchasing in each policy scenario at time steps 52 and 208, stratified by lower versus higher income strata. The left panel shows results from the pre-transition scenario in which UPF purchasing is greater in households with high income and the right panel shows results from the post-transition scenario. Generally, the magnitude of effects of most policies are similar but not the same for each of the income strata. For example, policy scenarios in the pre-transition that include a labeling policy achieve a greater reduction among the high-income strata, which virtually eliminates the difference between income strata observed at baseline. In contrast, baseline differences between income strata in the post-transition scenario generally remain unchanged or become more pronounced after policy implementation. To facilitate these comparisons, in Figure 3 we show the absolute difference in UPF purchasing between the low- and high-income strata at step 208. In the pre-transition scenario, the absolute difference in UPF purchasing between the higher and lower income strata is smaller post- versus pre-implementation of almost all policies. In the strongest policy combinations (i.e., those that include labeling and a 50% tax), the absolute difference in UPF purchasing between those in the middle/upper versus lower income strata are nearly eliminated. The reason is that the tax has a large effect among both groups, but the labeling policy has the largest effect among the group with the highest level of UPF purchasing.

In the post-transition scenario, the absolute difference between income strata generally stays the same or shrinks following implementation of each policy. However, the relative difference between income strata is largely unchanged or gets bigger. For example, prior to policy implementation, those in the higher income stratum consume 138 fewer kcal per week than those in the lower stratum, which equates to a difference of 4.6% between strata. Following implementation of the labeling policy, the difference between groups is 122 kcal per week. However, because consumption in both groups has fallen, the relative difference between strata has actually increased to about 5.4%. In the “all max” policy, the absolute difference between strata decreases to 66 kcal per week. Given the very large decrease in absolute UPF purchasing among both groups, however, this translates to a relative difference between groups of 7.8%. In Supplemental Tables 1 and 2, we present similar results by educational attainment strata.

## Discussion

We report results from an agent-based simulation model of policies to reduce UPF consumption in Latin America. Complex systems simulations – similar to the framework we presented in this study -- can complement both *in vivo* evaluations and previous simulation studies. First, the simulation approach enabled us to consider the effects of policies that have not yet been implemented in a given country, and for which there is no empirical record. Second, we considered the effects of multiple combinations of policies. An important way that the study complements *in vivo* studies and existing simulation research is by examining the effects of counter-advertising campaigns funded by the food and beverage industry in response to efforts to pass UPF tax and labeling policies.^(44, 45)^ One of the consequences of these counter-advertising campaigns is that increases in UPF consumption caused by aggressive industry advertising may partially offset the reductions achieved by tax and labeling policies. Disentangling these effects is important for understanding the effectiveness of these policies, but difficult with *in vivo* methods because the policies and counter-advertising are often implemented with overlapping timing and reach. To address this, we simulated scenarios in which the food and beverage industry respond to taxes and/or labeling by increasing levels of UPF advertising. The study also differs from prior simulation studies in its examination of the equity implications of different policy combinations, which depend upon pre-existing social patterns in UPF purchasing, heterogeneous policy effects, and UPF-related social norms and social influence.^(46, 47)^

We explored policies among a virtual population with levels of income, education, UPF purchasing, and UPF prices similar to those in Mexico, but the model is relevant and could be adapted to other Latin American countries. The model is most informative when thought of as a policy laboratory to evaluate the effects of implementing new policies or combining policies at different levels of intensity. Comparing policy options can be valuable for policymakers, as the political windows to pass major policies are often short and it may be difficult to replace or adjust ineffective policies. Similarly, policymakers often face constraints on resources and political capital. Generally, simulation studies can provide needed evidence for policies that can help policymakers choose between and justify policy choices.

Using input data from an evaluation study following the labeling law implemented in Chile and UPF price elasticities from multiple Latin American countries,^(15, 36, 37, 39)^ our results suggest that implementing a labeling law in a population similar to that in Mexico could reduce UPF purchasing by an amount that is roughly equivalent to that produced by a 20% tax. The model also suggests that moderate taxation and labeling policies produce effects that would require extremely large increases in industry advertising to replicate or offset. There are two implications of this finding: First, increases in industry advertising prior to and following implementation of UPF taxes, labeling, and other policies have likely not been large enough to offset policy effects,^(44, 45)^ but may have led to moderately attenuated estimates of policies’ effects. This is an insight generated by the model, as the effect of counter advertising is difficult to account for in *in vivo* studies because advertising increases typically occur at the same time as policy implementation. A second implication is that, if faced with limited political capital, policymakers should concentrate on taxes and labeling rather than policies limiting UPF advertising towards adults. Limits on marketing towards children – which have been proposed or implemented in several countries – may be more effective.

The model is also useful for understanding how different policy combinations are likely to affect social patterns in UPF consumption. International evidence suggests that countries may undergo a social transition in which the highest levels of UPF purchasing switch from higher-income to lower-income populations as a country’s income distribution shifts upwards.^(21)^ Some countries in Latin America may have recently gone through this transition, while others are still in the pre-transition stage. For example, a Chilean study using data from 2010 found that UPF consumption was highest in households in higher socioeconomic strata (i.e., the pre-transition pattern),^(5)^ but a later study found that by 2015 the highest levels of UPF consumption were among household in the lowest strata (i.e., the post-transition pattern).^(15)^ Our findings highlight that labeling policies – which have a larger impact among those with higher-levels of education – reduce differences between social strata in pre-transition contexts (because they reduce consumption in the higher income groups who have the highest consumption), but widens differences in post-transition contexts.^(15)^ A promising approach may be to combine tax and labeling policies, since taxes have a larger effect on households in the lower socioeconomic strata and labeling has the greatest effect on the highest socioeconomic strata.

As with any simulation study, a limitation is that the insights generated are tied to the model structure and parameters. The model is agnostic as to the specific mechanisms via which UPF prices, labeling, and advertising affect UPF purchasing – rather, we identified effect estimates from relevant evaluation studies and the extant literature. Use of these effect estimates is both a strength and limitation of the study: it is a strength because we do not need to specify a specific causal structure via which policies achieve their effects, which may lessen the risk of bias from misspecification. However, an assumption inherent to this approach is that the effect sizes and elasticity estimates we used are valid and relevant to the Latin American context. Generally, studies of the own-price elasticity of multiple UPF products in multiple Latin American countries produced fairly similar estimates (i.e., -1 to -1.4).^(36–39)^

Though few cities or countries have implemented mandatory UPF labeling laws, the recent evaluation study from Chile is an ideal model policy because it is likely similar to what would be passed in other Latin American countries and because the authors reported separate effect estimates by level of educational attainment. The stratified effects enabled us to assess how a labeling policy could reduce or exacerbate existing differences in UPF purchasing between social strata. Notably, the effect sizes are comparable to estimates from a meta-analysis of smaller-scale experimental studies conducted in the U.S. and Europe.^(15)^ We were unable to identify a study of advertising effectiveness (i.e., the effect of exposure to advertising on food purchasing) in the Latin American context, either for food advertising or generally. We used an estimate of advertising elasticity from a meta-analysis of studies of advertising effectiveness.^(41)^ The advertising elasticity implies a level of advertising ineffectiveness that our team found surprising given that companies spend billions of dollars per year on advertising. Nonetheless, the low advertising elasticity value is consistent with other values reported in the literature, including a recent study of advertising effects across a large number of products.^(48)^ A caveat is that these studies were based on effect of television advertising, and point-of-purchase advertising, billboards, and other forms of advertising may be more effective. Given the pervasive level of UPF advertising in Latin American countries, an area for future research is evaluating the effect of different forms of UPF advertising on purchasing.

A further consideration is that the purpose of the model is to explore the effects of specific policy levers on UPF purchasing and does not include all drivers of UPF purchasing. Because we did not examine policies to change healthy food access, for example, the model is aspatial and does not consider agents’ proximity to healthy and unhealthy food retailers. Similarly, we did not consider the effects of product reformulation in response to taxes, labels, and other policies.^(49)^

In this study, we presented a framework and virtual laboratory for exploring how available public policy levers can be used – both alone and in combination – to address high levels of UPF purchasing in Latin American countries at different stages of the social transition in UPF purchasing and with different UPF prices, purchasing levels, and social characteristics. Our results using UPF price, purchasing, and social data from Mexico suggest that differential effectiveness of policies can either reduce or exacerbate differences in UPF purchasing between socioeconomic strata. Given evidence suggesting that countries in Latin America are at different stages of the social transition in UPF purchasing, policymakers should consider the equity implications of policy as part of the planning process.

## Supplementary Material

Supplemental Information

## Figures and Tables

**Table 1:** Network characteristics

Network	N	Number of links	Proportion of links / nodes	Average degree	Mean path length	Local clustering coefficient	Global clustering coefficient
Typical small world	800	2337	2.921	5.842	4.3068	0.007	
ABM network	1000	2744	2.744	5.47	4.24	0.0127	0.0023

Note: characteristics of the typical network are from Chen (2019)^(50)^

## References

[1] Monteiro CA, Moubarac JC, Cannon G (2013). Ultra-processed products are becoming dominant in the global food system. Obesity reviews : an official journal of the International Association for the Study of Obesity.

[2] Monteiro CA, Moubarac J-C, Levy RB (2018). Household availability of ultra-processed foods and obesity in nineteen European countries. Public Health Nutrition.

[3] Marrón-Ponce JA, Sánchez-Pimienta TG, Louzada M (2018). Energy contribution of NOVA food groups and sociodemographic determinants of ultra-processed food consumption in the Mexican population. Public Health Nutr.

[4] Khandpur N, Cediel G, Obando DA (2020). Sociodemographic factors associated with the consumption of ultra-processed foods in Colombia. Revista de saude publica.

[5] Cediel G, Reyes M, da Costa Louzada ML (2018). Ultra-processed foods and added sugars in the Chilean diet. Public Health Nutr.

[6] Monteiro CA, Cannon G, Levy RB (2019). Ultra-processed foods: what they are and how to identify them. Public Health Nutr.

[7] Monteiro CA, Cannon G, Moubarac JC (2018). The UN Decade of Nutrition, the NOVA food classification and the trouble with ultra-processing. Public Health Nutr.

[8] Monteiro CA, Levy RB, Claro RM (2010). Increasing consumption of ultraprocessed foods and likely impact on human health: evidence from Brazil. Public Health Nutrition.

[9] Marrón-Ponce JA, Flores M, Cediel G (2019). Associations between consumption of ultra-processed foods and intake of nutrients related to chronic non-communicable diseases in Mexico. Journal of the Academy of Nutrition and Dietetics.

[10] Costa CS, Del-Ponte B, Assunção MCF (2018). Consumption of ultra-processed foods and body fat during childhood and adolescence: a systematic review. Public Health Nutr.

[11] Canella DS, Levy RB, Martins APB (2014). Ultra-Processed Food Products and Obesity in Brazilian Households (2008–2009). PLOS ONE.

[12] Colchero MA, Rivera-Dommarco J, Popkin BM (2017). In Mexico, evidence of sustained consumer response two years after implementing a sugar-sweetened beverage tax. Health Affairs.

[13] Taillie LS, Rivera JA, Popkin BM (2017). Do high vs. low purchasers respond differently to a nonessential energy-dense food tax? Two-year evaluation of Mexico’s 8% nonessential food tax. Preventive medicine.

[14] Caro JC, Ng SW, Taillie LS (2017). Designing a tax to discourage unhealthy food and beverage purchases: The case of Chile. Food Policy.

[15] Taillie LS, Reyes M, Colchero MA (2020). An evaluation of Chile’s Law of Food Labeling and Advertising on sugar-sweetened beverage purchases from 2015 to 2017: A before-and-after study. PLOS Medicine.

[16] Reyes M, Garmendia ML, Olivares S (2019). Development of the Chilean front-of-package food warning label. BMC Public Health.

[17] Soares Guimarães J, Mais LA, Marrocos Leite FH (2020). Ultra-processed food and beverage advertising on Brazilian television by International Network for Food and Obesity/Non-Communicable Diseases Research, Monitoring and Action Support benchmark. Public Health Nutrition.

[18] Allemandi L, Castronuovo L, Tiscornia MV (2018). Food advertising on Argentinean television: are ultra-processed foods in the lead?. Public Health Nutrition.

[19] Correa T, Reyes M, Taillie LS (2020). Food Advertising on Television Before and After a National Unhealthy Food Marketing Regulation in Chile, 2016–2017. American Journal of Public Health.

[20] Colchero MA, Popkin BM, Rivera JA (2016). Beverage purchases from stores in Mexico under the excise tax on sugar sweetened beverages: observational study. BMJ.

[21] Baker P, Machado P, Santos T (2020). Ultra-processed foods and the nutrition transition: Global, regional and national trends, food systems transformations and political economy drivers. Obesity Reviews.

[22] Hammond RA, Ornstein JT, Fellows LK (2012). A model of food reward learning with dynamic reward exposure. Frontiers in Computational Neuroscience.

[23] Christakis NA, Fowler JH (2007). The spread of obesity in a large social network over 32 years. New England Journal of Medicine.

[24] McPherson M, Smith-Lovin L, Cook JM (2001). Birds of a Feather: Homophily in Social Networks. Annual Review of Sociology.

[25] Wright EO (1997). Class counts: Comparative studies in class analysis.

[26] Marsden PV (1987). Core discussion networks of Americans. American sociological review.

[27] Wilenski U (1999). NetLogo. Evanston, IL: Center for Connected Learning and Computer-Based Modeling, Northwestern University.

[28] Hall KD, Sacks G, Chandramohan D (2011). Quantification of the effect of energy imbalance on bodyweight. Lancet (London, England).

[29] Confederação Nacional de Dirigentes Lojistas (2016). Facetas da mulher Brasileira: mercado de consumo.

[30] Lelis CT, Teixeira KMD, Silva NMd (2012). A inserção feminina no mercado de trabalho e suas implicações para os hábitos alimentares da mulher e de sua família. Saúde em Debate.

[31] OECD (2019). Under Pressure: The Squeezed Middle Class.

[32] Marrón-Ponce J, Tolentino-Mayo L, Hernández-F M (2019). Trends in ultraprocessed food purchases from 1984 to 2016 in Mexican households. Nutrients.

[33] Verbrugge LM (1983). A research note on adult friendship contact: a dyadic perspective. Soc F.

[34] Hammond RA (2010). Social influence and obesity. Current Opinion in Endocrinology, Diabetes and Obesity.

[35] Hammond RA, Ornstein JT (2014). A model of social influence on body mass index. Annals of the New York Academy of Sciences.

[36] Chacon V, Paraje G, Barnoya J (2018). Own-price, cross-price, and expenditure elasticities on sugar-sweetened beverages in Guatemala. PLOS ONE.

[37] Guerrero-López CM, Unar-Munguía M, Colchero MA (2017). Price elasticity of the demand for soft drinks, other sugar-sweetened beverages and energy dense food in Chile. BMC public health.

[38] Paraje G (2016). The effect of price and socio-economic level on the consumption of sugar-sweetened beverages (SSB): the case of Ecuador. PLoS One.

[39] Colchero MA, Salgado JC, Unar-Munguía M (2015). Price elasticity of the demand for sugar sweetened beverages and soft drinks in Mexico. Economics & Human Biology.

[40] Colchero MA, Guerrero-López CM, Molina M (2016). Beverages Sales in Mexico before and after Implementation of a Sugar Sweetened Beverage Tax. PLOS ONE.

[41] Hu Y, Lodish LM, Krieger AM (2007). An analysis of real world TV advertising tests: A 15-year update. Journal of Advertising Research.

[42] Global Food Research Program (2021). Sugary drink taxes around the world.

[43] Cawley J, Frisvold D, Hill A (2019). The impact of the Philadelphia beverage tax on purchases and consumption by adults and children. Journal of Health Economics.

[44] Barquera S, Campos I, Rivera JA (2013). Mexico attempts to tackle obesity: the process, results, push backs and future challenges. Obesity Reviews.

[45] Backholer K, Blake M, Vandevijvere S (2017). Sugar-sweetened beverage taxation: an update on the year that was 2017. Public health nutrition.

[46] Basto-Abreu A, Barrientos-Gutiérrez T, Vidaña-Pérez D (2019). Cost-Effectiveness Of The Sugar-Sweetened Beverage Excise Tax In Mexico. Health Affairs.

[47] Liu SY, Osgood N, Gao Q (2016). Systems simulation model for assessing the sustainability and synergistic impacts of sugar-sweetened beverages tax and revenue recycling on childhood obesity prevention. Journal of the Operational Research Society.

[48] Shapiro B, Hitsch GJ, Tuchman A (2020). Generalizable and robust TV advertising effects. NBER Working Paper.

[49] Reyes M, Smith Taillie L, Popkin B (2020). Changes in the amount of nutrient of packaged foods and beverages after the initial implementation of the Chilean Law of Food Labelling and Advertising: A nonexperimental prospective study. PLOS Medicine.

[50] Chen Z (2019). An Agent-Based Model for Information Diffusion over Online Social Networks. Papers in Applied Geography.

