# Potential impacts of policies to reduce purchasing of ultra-processed foods in Mexico at different stages of the social transition: an agent-based modelling approach

**DOI:** 10.1017/S1368980021004833

**Published:** 2022-06

**Authors:** Brent A Langellier, Ivana Stankov, Ross A Hammond, Usama Bilal, Amy H Auchincloss, Tonatiuh Barrientos-Gutierrez, Leticia de Oliveira Cardoso, Ana V Diez Roux

**Affiliations:** 1 Department of Health Management and Policy, Dornsife School of Public Health, Drexel University, 3215 Market St, Office 356, Philadelphia, PA 19104, USA; 2 Urban Health Collaborative, Dornsife School of Public Health, Drexel University, Philadelphia, PA, USA; 3 UniSA Allied Health and Human Performance, University of South Australia, Adelaide, South Australia, Australia; 4 Center on Social Dynamics & Policy, Brookings Institution, Washington, DC, USA; 5 Public Health and Social Policy, Washington University in St. Louis, St. Louis, MO, USA; 6 Department of Epidemiology and Biostatistics, Dornsife School of Public Health, Drexel University, Philadelphia, PA, USA; 7 Center for Population Health Research, National Institute of Public Health, Cuernavaca, México; 8 Oswaldo Cruz Foundation, Rio de Janeiro, Brazil

**Keywords:** Food policy, Diet, Complex systems, Social determinants of health, Simulation

## Abstract

**Objectives:**

To develop a simulation framework for assessing how combinations of taxes, nutrition warning labels and advertising levels could affect purchasing of ultra-processed foods (UPF) in Latin American countries and to understand whether policies reinforce or reduce pre-existing social disparities in UPF consumption.

**Design:**

We developed an agent-based simulation model using international evidence regarding the effect of price, nutrition warning labels and advertising on UPF purchasing.

**Setting:**

We estimated policy effects in scenarios representing two stages of the ‘social transition’ in UPF purchasing: (1) a pre-transition scenario, where UPF purchasing is higher among high-income households, similar to patterns in Mexico; and (2) a post-transition scenario where UPF purchasing is highest among low-income households, similar to patterns in Chile.

**Participants:**

A population of 1000 individual agents with levels of age, income, educational attainment and UPF purchasing similar to adult women in Mexico.

**Results:**

A 20 % tax would decrease purchasing by 24 % relative to baseline in both the pre- and post-transition scenarios, an effect that is similar in magnitude to that of a nutrition warning label policy. A 50 % advertising increase or decrease had a comparatively small effect. Nutrition warning labels were most effective among those with higher levels of educational attainment. Labelling reduced inequities in the pre-transition scenario (i.e. highest UPF purchasing among the highest socio-economic group) but widened inequities in the post-transition scenario.

**Conclusions:**

Effective policy levers are available to reduce UPF purchasing, but policymakers should anticipate that equity impacts will differ depending on existing social patterns in UPF purchasing.

Ultra-processed foods (UPF) are an increasingly dominant part of the global food system^(1)^, and their availability and consumption have increased in most countries and regions^(2–5)^. UPF are foods that have been developed via ‘fractioning of whole foods into substances, chemical modifications of these substances, assembly of unmodified and modified food substances, frequent use of cosmetic additives and sophisticated packaging’^(6)^. Engineered to maximise profit margins, convenience, shelf stability and palatability relative to unprocessed or minimally processed foods^(7)^, they tend to have more added sugar, more saturated fat, more Na, less fibre, less micronutrients and much higher energy density^(8,9)^. UPF consumption is positively associated with development of obesity among youth and adults^(10,11)^.

In many Latin American countries, calories from UPF contribute 20–30 % or more to total energy intake^(3,5)^. Latin American countries have been at the forefront of using policy levers to address rising UPF consumption, including UPF taxes, front-of-package nutrition warning labels and advertising restrictions^(6,8)^. In 2014, Mexico implemented an 8 % tax on non-essential energy-dense foods and a peso-per-litre (roughly equivalent to 10 %) tax on sugar-sweetened beverages^(12,13)^. Purchasing of non-essential energy-dense foods fell 7·4 % 2 years after implementation of the tax and purchasing of taxed beverages fell 9·7 %^(12,13)^. Also in 2014, Chile increased an existing beverage tax from 13 % to 18 % for beverages high in sugar and reduced the tax rate from 13 % to 10 % for beverages low in sugar^(14)^. As part of a front-of-package labelling and advertising law passed in 2016, Chile was the first country in the world to mandate front-of-package nutrition labels on energy-dense foods, with other countries (including Peru and Uruguay) subsequently adopting their own labelling policies^(15,16)^. Two years after implementation of the Chilean law, purchasing of beverages high in added sugar decreased by 23·7 %^(15)^. Several countries in the region have also passed policies to address high levels of UPF advertising^(17–19)^. For example, 14 % of advertisements on the major ‘free to air’ television channels in Brazil are food-related, 91 % of which are for UPF products^(17)^.

Evaluations of these vanguard policies in Latin America suggest that policy levers can meaningfully reduce UPF consumption at the population level. A remaining question is how policies can be used without creating or exacerbating existing inequities in UPF consumption. For example, evaluation data from Chile suggest that the labelling law had larger effects among individuals with high educational attainment who had lower consumption levels even prior to the tax^(15)^. In contrast, the strongest impacts from the taxes implemented in Mexico were among households in the lower socio-economic strata^(20)^.

The equity implications of the UPF reduction policies a country implements may depend on existing social patterns in UPF consumption, which vary between countries. UPF consumption is inversely associated with socio-economic status in higher-income countries, but the reverse is true in lower- and middle-income countries^(21)^. Baker and colleagues (2020) suggest that a ‘social transition’ takes place as a country’s income distribution shifts upwards^(21)^. In the first stage, the highest levels of UPF consumption are among individuals in the highest socio-economic strata. As the income distribution shifts upwards, however, the highest levels of UPF consumption transition to those in the lower socio-economic groups. Different Latin American countries may be in each stage. For example, higher-income households purchase more UPF than lower-income households in Mexico^(3)^, but the reverse is true in Chile^(15)^.

Like many health behaviours, dietary choices are socially determined based on community-level social norms and social influence between peers, family members and other close social contacts^(22,23)^. Policies to reduce UPF consumption will need to reverse secular trends that have made high levels of UPF consumption normative in most Latin American countries. Social influence on dietary choices likely contributes to the production and persistence of social inequities in UPF purchasing, particularly given international research suggesting a high degree of social homophily – meaning that strong social ties are most commonly formed between individuals with similar social characteristics, including age, educational attainment and income^(24–26)^. In combination, social homophily and social influence are key mechanisms that contribute to the production and persistence of inequities between groups; these inequities can either be reduced or exacerbated by policies that have differential effects across social groups^(15,20)^.

In this study, we report the results of an agent-based model developed to understand the relative effectiveness of varying combinations of UPF tax, labelling and advertising policies on UPF purchasing. We examine policy effects among a population of agents with social characteristics (age, income and educational attainment) that loosely represent female food purchasers in Mexican households. We consider policy effects in two broad scenarios: the first represents countries in which UPF purchasing is highest among high-income households (i.e. a pre-social transition in UPF purchasing similar to patterns observed in Mexico)^(21)^. In the second scenario, UPF purchasing is highest among low-income households (i.e. a post-social transition scenario that represents a plausible future scenario for Mexico and is qualitatively similar to patterns observed in Chile and in high-income countries). For each policy combination, we report the population-level effects, as well as stratified effects by income and educational attainment that are helpful for understanding how policies either reduce or exacerbate differences in UPF purchasing between socio-economic strata.

## Methods

### Agent population and properties

We coded the model in NetLogo^(27)^. Full details regarding model design, data sources and effect parameters are in the model sketch in Appendix 2. We simulated weekly UPF purchasing, measured in kilocalories (kcal) purchased per week, among a virtual population of 1000 individual agents with levels of age, income and educational attainment similar to those of adult women in Mexico. We selected weekly UPF purchasing in kcal because they are a widely used measure of energy intake, an important input to energy balance and weight change^(28)^, and because they can be calculated across different types of UPF items (e.g. sweetened beverages, candy). We limited the population to adult women for three reasons: (1) women typically are the primary food purchasers in their household^(29,30)^; (2) adults and children have different food purchasing and consumption patterns, so focusing on adult women simplifies the parameters (e.g. distribution of UPF purchasing) and behaviour rules in the model (e.g. social signal)^(3,4)^; and (3) research on social homophily suggests that close social ties are most common among those of the same gender – including multiple genders in the model would unnecessarily complicate the social network formation and social signal, described below^(24)^.

Upon initialisation of the model, each agent was assigned three characteristics that are important for food behaviours and social network formation: age group (young, middle-aged and older), income and educational attainment. The age categories are intended to make a qualitative distinction between women in different life stages. We assigned one-quarter of agents to the younger and older age categories and 50 % to the middle-aged category. Based on data from the 2016 Mexican Survey of Household Income and Spending (ENIGH), the categories generally correspond to women in the following age ranges: (1) 20 to 30 years; (2) 31 to 50 years and (3) >50 years. We used data from the 2016 ENIGH to inform the initial distributions of household income and educational attainment. Specifically, 27 % of agents were assigned to the high-education category, representing at least a high school education, and 73 % to the low education category, representing less than high school. We assigned each agent a continuous household income drawn from separate log-normal distributions for those with low education (mean = 889 pesos/week, SD = 911) and high education (mean = 2044, SD = 2225). We then assigned agents with income above a threshold value (1890 pesos/week) to a high-income category and those below to the low-income category^(31)^. We assigned both continuous and categorical income because the former is used in calculating relative UPF prices and the latter in social network formation. Agent properties and the data sources used to inform their distributions are summarised in Table A-1 in Appendix 2.

### Baseline scenarios: ultra-processed food purchasing

We examined policy effects under two baseline scenarios that represent pre- *v*. post-stages in the social transition in UPF purchasing^(21)^. For both scenarios, we used data from Marrón-Ponce *et al*. (2019) to inform the initial distribution of UPF purchasing^(32)^. Specifically, we calibrated the mean values among low- and high-income agents to reproduce the mean weekly purchasing among all household reported by Marrón-Ponce, which was 3033 kcal/week. We calibrated the values for each income group because they were not reported by Marrón-Ponce or elsewhere. In the pre-transition scenario, the calibrated values at which we set mean UPF purchasing were 3446 kcal/week for high-income agents and 2966 kcal/week for low-income agents. This scenario is similar to existing patterns in UPF purchasing in Mexico, as reported by Marrón-Ponce. In the post-transition scenario, we set the mean calibrated UPF purchasing at 2620 kcal/week for high-income agents and at 3100 kcal/week for low-income agents. This is a counterfactual scenario for pre-transition countries, including Mexico, but is generally similar to patterns in post-transition countries, such as Chile^(15)^.

### Social network

Agents in the model were embedded in a small world network (with average node degree of 5·47), where the connections between agents represented close friendships between women in different households. Consistent with *in vivo* studies of social homophily^(24–26,33)^, agents were more likely to be connected to other agents with similar age, income and educational attainment levels. Agents had at least three and a maximum of fifty social connections. As shown in Table 1, we set the social network parameters to reproduce network characteristics similar to that reported in Chen (2019).

**Table 1:** Network characteristics

Network	*n*	Number of links	Proportion of links/nodes	Average degree	Mean path length	Local clustering coefficient	Global clustering coefficient
Typical small world	800	2337	2·921	5·842	4·3068	0·007	
Agent-based model network	1000	2744	2·744	5·47	4·24	0·0127	0·0023

Characteristics of the typical network are from Chen (2019)^(50)^.

### Updates to ultra-processed food purchasing

Every time step, each agent in the model made a series of adjustments to her UPF purchasing level. The first adjustment represented the effects of a social signal and social norms^(34,35)^. The social signal can be thought of as representing processes of social learning and peer influence that occur between friends and family members^(34)^. Social norms can be thought of as individuals’ desire to adhere to group-level norms among people with similar levels of age, income and educational attainment.

For each adjustment, the agent calculated whether her own level of weekly UPF purchasing differed from the average among her social network by more than 50 kcal/week. The 50 kcal threshold represents uncertainty in people’s knowledge of the true levels of UPF purchasing of their friends. If the difference was less than the threshold, the agent made no adjustment. If the difference was greater than the threshold, she shifted towards the social signal by a fractional amount (i.e. 10 % of the difference). The same process was repeated for social norms, but each agent compared her purchasing to the average purchasing of all agents with the same levels of age (i.e. younger, middle-aged, older), income (i.e. lower and higher) and education status (i.e. lower and higher).

The second type of update that agents made was in response to UPF policy changes. We used external data to inform the magnitude of these updates. The model calculated UPF purchasing in response to price changes based on the own-price elasticity of UPF. We set the own-price elasticity of UPF to –1·2 (i.e. a 1 % increase in the price of UPF could be expected to produce a 1·2 % decline in purchasing) based on studies of price elasticities of sugar-sweetened beverages in several Latin American countries, all of which range from –1·0 to –1·4^(36–39)^. Notably, this range of values is within the range of effects observed at 2 years post-implementation of the UPF taxes implemented in Mexico (i.e. a 10–12 % reduction at 2 years post-implementation of ∼10 % tax)^(13,20,40)^. Based on the main evaluation study of the Chilean labelling policy^(15)^, we set agents’ sensitivity to labelling (i.e. the difference in purchasing caused by a switch from no label to a front-of-package label) to −22 % for low-education agents and −29 % for high-education agents. We set each agent’s sensitivity to advertising (i.e. the advertising elasticity) to 0·113 based on Hu *et al*. (2007)^(41)^.This means that a 1 % increase in advertising translates to a 0·113 % increase in purchasing. In simulation scenarios with multiple policy interventions, the change in purchasing from each of the policies is summed.

### Calibration

Calibration is an iterative process of making adjustments to parameter values such that model outcomes align with specified values or calibration targets. We used calibration experiments to set the values of four unknown parameters: (1) resistance to conforming to the social signal and social norms; (2) the relative importance of social similarity (e.g. same-age group) in constructing the social network; (3) the baseline level of UPF purchasing in low-income agents and (4) the baseline level of UPF purchasing in high-income agents. The first two parameters were the same in the pre- and post-social transition scenarios, and only the third and fourth differed. The calibration target we used was that the average, equilibrium state weekly household UPF purchasing at the population level must be within 5 kcal of the calibration target of 3033 kcal/week based on findings reported in Marrón-Ponce *et al.* (2019)^(32)^. In the pre-social transition scenario, we required that low-income households had lower weekly UPF consumption than high-income households, and vice versa for the post-social transition scenario. We also required that the calibrated parameters produce unique distributions of weekly household UPF purchasing by income and that these distributions remained unique (i.e. did not fully converge) as the model ran.

### Policy counterfactuals

We used the agent-based model to examine how UPF purchasing would be affected by a UPF labelling policy and UPF taxes of 8 % (actual junk food tax in Mexico), 20 % (beverage tax level considered in Mexico but not passed) and 50 % (counterfactual ‘high tax’ scenario). Several countries in the Arabian peninsula have implemented taxes of 50 % or more on sweetened beverages and excises taxes implemented in several local areas in the USA (e.g. 1·5, 1·75 and 2·0 cents per ounce in Philadelphia, Seattle and Boulder, respectively) equate to over 50 % for some products^(42,43)^. These examples suggest that, though taxes in this amount have not been implemented in Latin American countries, they are of a level that could plausibly be considered by policymakers at both the local and national level. We also examined how policy effects would be impacted by increases in UPF advertising of 25 % and 50 %, representing industry responses to labelling and tax policies, as well as similar decreases in UPF advertising, representing policy restrictions on advertising. For both the pre- and post-social transition scenarios, we ran iterations of the model with no policy, with each policy implemented alone and with multiple policy combinations.

The model ran in discrete time, with each time step representing 1 week. We compared mean UPF purchasing in each scenario after 208 time steps (i.e. 4 years), not including a burn-in period of 100 time steps. The burn-in period allowed each agent to update her food purchasing until the model reached a stable state that aligned with population-level UPF purchasing trends (calibration target) reported by Marrón-Ponce *et al.* (2019)^(32)^. We implemented policies 1 year into the simulation (i.e. after 52 time steps) and ran each scenario 200 times to account for random variation. This number of runs was determined through sensitivity analyses which suggested that only relatively small variations in weekly UPF purchasing were observed for simulations higher than 200 runs (see Appendix Fig. 2-A).

## Results

**Fig. 1:**
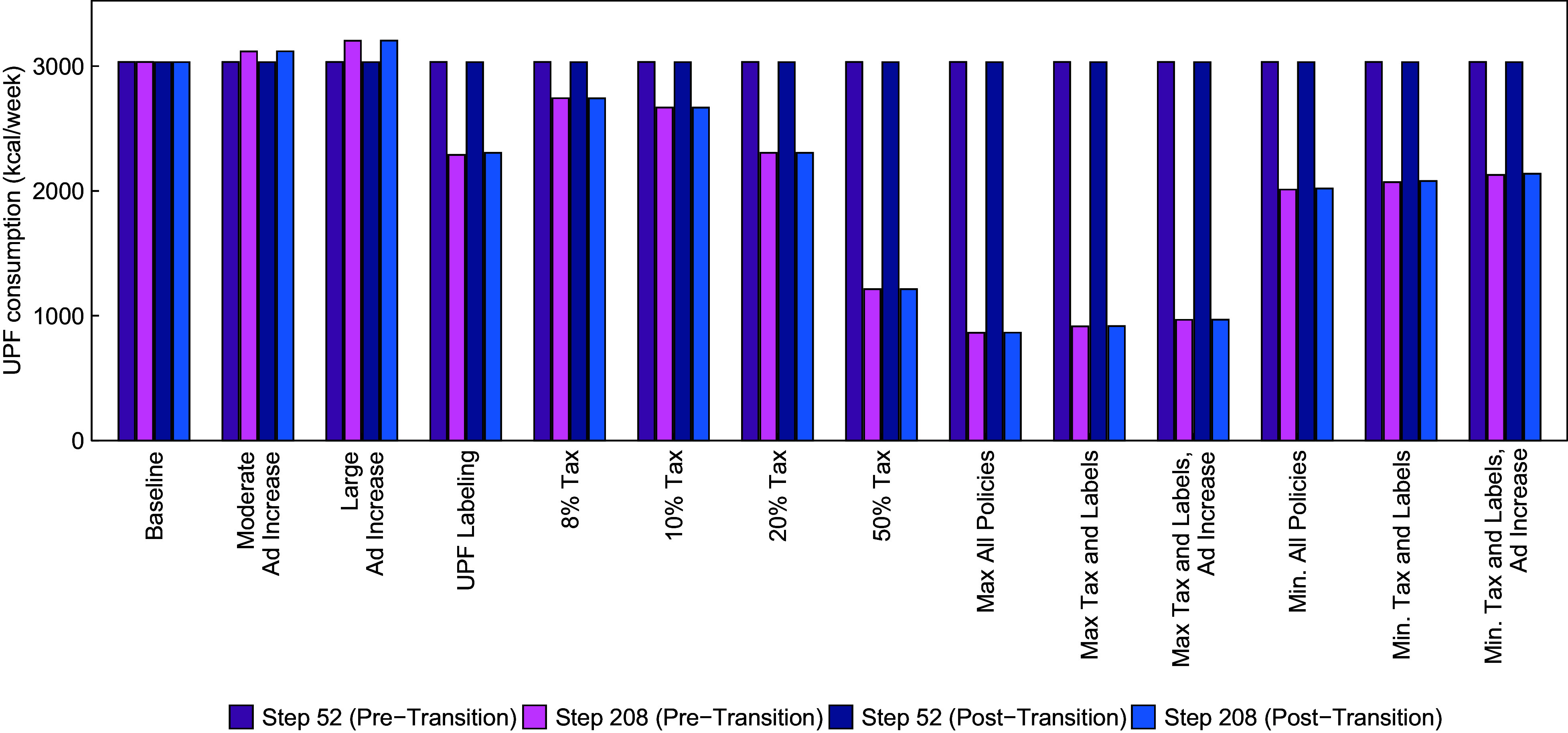
Mean weekly purchasing of ultra-processed foods in kcal/week at time steps 52 (just prior to policy implementation) and 208 (equivalent to 3 years post-implementation), by policy scenario and stage of the social transition in UPF purchasing

In Fig. 1, we show mean UPF purchasing under different policy combinations at time steps 52 (just prior to policy implementation) and 208 (∼3 years after implementation) in both the pre- and post-social transition scenarios. In both the post- and pre-social transition scenarios, mean UPF purchasing demonstrated good fit to the average weekly UPF purchasing (i.e. 3033 kcal/week) estimated by Marrón-Ponce *et al.* (2019). Generally, the population-level effects of each policy were similar between the post- and pre-transition scenarios.

Among the policies implemented alone, the 50 % tax produced the largest decrease in UPF purchasing, a decrease of about 60 % relative to the baseline in both the pre- and post-transition scenarios. The labelling policy and the 20 % tax each had similar effects when implemented alone, decreasing UPF purchasing by about 24 %. Notably, the effect of a 50 % change in advertising was much smaller than the tax or labelling policies – for example, a 50 % increase in advertising resulted in an increase in UPF purchasing by 6 %. Among policies implemented in combination, the scenario that included a 50 % tax, labelling and a 50 % reduction in advertising reduced UPF purchasing by 72 %. This scenario assumes that the amount of advertising promoting UPF purchasing would be reduced, likely through a policy change that limited or taxed industry advertising. Without any change in advertising, the 50 % tax and label policy still decreased UPF purchasing by 70 %. Even if industry responded to the tax and labelling policies by *increasing* advertising levels by 50 %, UPF purchasing would still be lower than the baseline scenario by 68 %.

The 8 % UPF tax, which resembles the junk food tax implemented in Mexico, decreased weekly UPF purchasing by about 10 % relative to pre-implementation levels. If Mexico were to also implement a UPF labelling policy, the model estimates a reduction in UPF purchasing by about 31 % (approximately 2000 kcal/week). Even if industry responded by increasing UPF advertising by 25 %, the reduction in UPF purchasing would be very similar (just under 30 % or approximately 2100 calories/week).

**Fig. 2:**
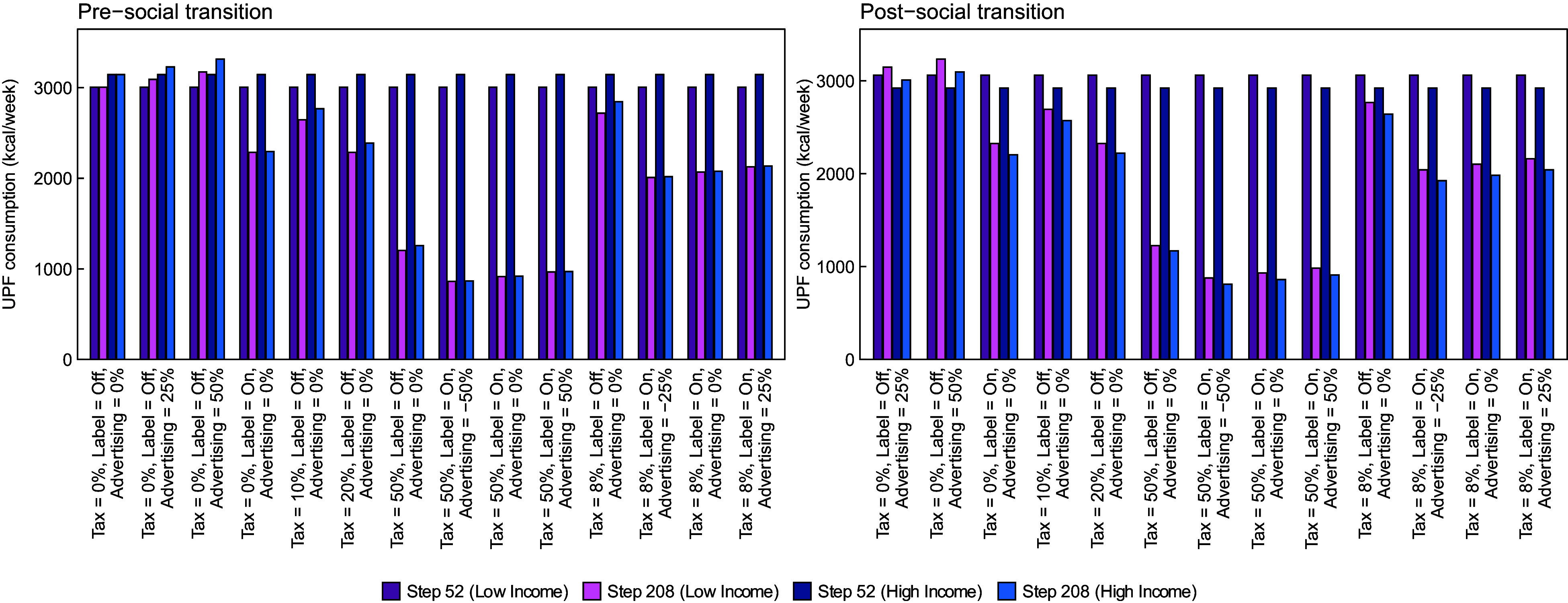
Mean weekly purchasing of ultra-processed foods in kcal/week by income strata, in a population in which UPF purchasing is greater in households with either higher income (pre-social transition, left panel) or lower income (post-social transition, left panel)

**Fig. 3:**
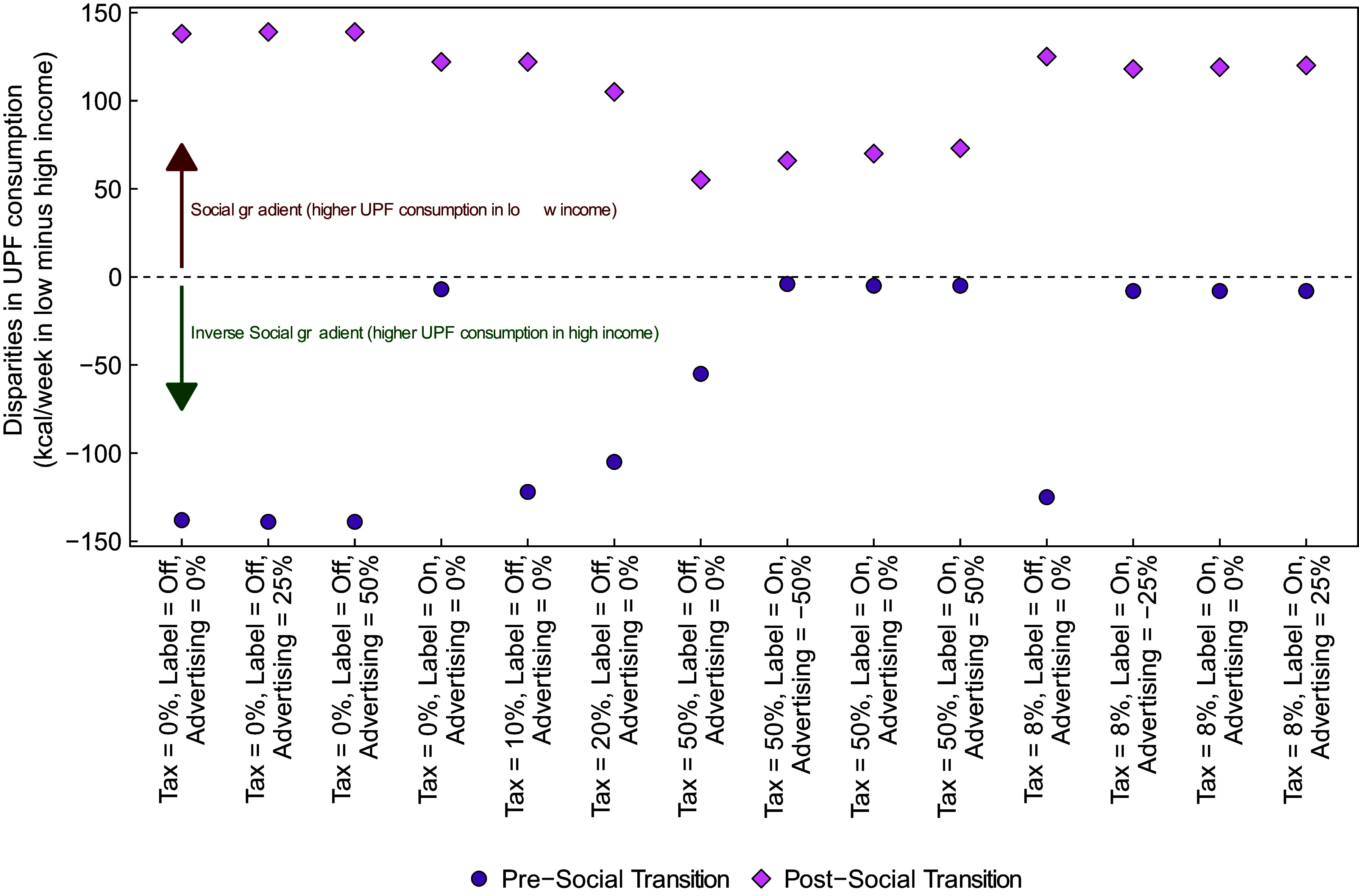
Difference in weekly purchasing of ultra-processed foods between the lower and higher-income strata at time step 208, by stage of the social transition

In Fig. 2, we report estimated UPF purchasing in each policy scenario at time steps 52 and 208, stratified by lower- *v*. higher-income strata. The left panel shows results from the pre-transition scenario in which UPF purchasing is greater in households with high income, and the right panel shows results from the post-transition scenario. Generally, the magnitude of effects of most policies is similar but not the same for each of the income strata. For example, policy scenarios in the pre-transition that include a labelling policy achieve a greater reduction among the high-income strata, which virtually eliminates the difference between income strata observed at baseline. In contrast, baseline differences between income strata in the post-transition scenario generally remain unchanged or become more pronounced after policy implementation. To facilitate these comparisons, in Fig. 3 we show the absolute difference in UPF purchasing between the low- and high-income strata at time step 208. In the pre-transition scenario, the absolute difference in UPF purchasing between the higher and lower-income strata is smaller post- *v*. pre-implementation of almost all policies. In the strongest policy combinations (i.e. those that include labelling and a 50 % tax), the absolute difference in UPF purchasing between those in the middle/upper *v*. lower-income strata is nearly eliminated. The reason is that the tax has a large effect among both groups, but the labelling policy has the largest effect among the group with the highest level of UPF purchasing.

In the post-transition scenario, the absolute difference between income strata generally stays the same or shrinks following implementation of each policy. However, the relative difference between income strata is largely unchanged or gets bigger. For example, prior to policy implementation, those in the higher-income stratum consume 138 fewer kcal/week than those in the lower stratum, which equates to a difference of 4·6 % between strata. Following implementation of the labelling policy, the difference between groups is 122 kcal/week. However, because consumption in both groups has fallen, the relative difference between strata has actually increased to about 5·4 %. In the ‘all max’ policy, the absolute difference between strata decreases to 66 kcal/week. Given the very large decrease in absolute UPF purchasing among both groups, however, this translates to a relative difference between groups of 7·8 %. In Supplemental Tables 1 and 2, we present similar results by educational attainment strata.

## Discussion

We report results from an agent-based simulation model of policies to reduce UPF consumption in Latin America. Complex systems simulations – similar to the framework we presented in this study – can complement both *in vivo* evaluations and previous simulation studies. First, the simulation approach enabled us to consider the effects of policies that have not yet been implemented in a given country and for which there is no empirical record. Second, we considered the effects of multiple combinations of policies. An important way that the study complements *in vivo* studies and existing simulation research is by examining the effects of counter-advertising campaigns funded by the food and beverage industry in response to efforts to pass UPF tax and labelling policies^(44,45)^. One of the consequences of these counter-advertising campaigns is that increases in UPF consumption caused by aggressive industry advertising may partially offset the reductions achieved by tax and labelling policies. Disentangling these effects is important for understanding the effectiveness of these policies, but difficult with *in vivo* methods because the policies and counter-advertising are often implemented with overlapping timing and reach. To address this, we simulated scenarios in which the food and beverage industry respond to taxes and/or labelling by increasing levels of UPF advertising. The study also differs from prior simulation studies in its examination of the equity implications of different policy combinations, which depend on pre-existing social patterns in UPF purchasing, heterogeneous policy effects and UPF-related social norms and social influence^(46,47)^.

We explored policies among a virtual population with levels of income, education, UPF purchasing and UPF prices similar to those in Mexico, but the model is relevant and could be adapted to other Latin American countries. The model is most informative when thought of as a policy laboratory to evaluate the effects of implementing new policies or combining policies at different levels of intensity. Comparing policy options can be valuable for policymakers, as the political windows to pass major policies are often short and it may be difficult to replace or adjust ineffective policies. Similarly, policymakers often face constraints on resources and political capital. Generally, simulation studies can provide needed evidence for policies that can help policymakers choose between and justify policy choices.

Using input data from an evaluation study following the labelling law implemented in Chile and UPF price elasticities from multiple Latin American countries^(15,36,37,39)^, our results suggest that implementing a labelling law in a population similar to that in Mexico could reduce UPF purchasing by an amount that is roughly equivalent to that produced by a 20 % tax. The model also suggests that moderate taxation and labelling policies produce effects that would require extremely large increases in industry advertising to replicate or offset. There are two implications of this finding: First, increases in industry advertising prior to and following implementation of UPF taxes, labelling and other policies have likely not been large enough to offset policy effects^(44,45)^ but may have led to moderately attenuated estimates of policies’ effects. This is an insight generated by the model, as the effect of counter-advertising is difficult to account for in *in vivo* studies because advertising increases typically occur at the same time as policy implementation. A second implication is that, if faced with limited political capital, policymakers should concentrate on taxes and labelling rather than policies limiting UPF advertising towards adults. Limits on marketing towards children – which have been proposed or implemented in several countries – may be more effective.

The model is also useful for understanding how different policy combinations are likely to affect social patterns in UPF consumption. International evidence suggests that countries may undergo a social transition in which the highest levels of UPF purchasing switch from higher-income to lower-income populations as a country’s income distribution shifts upwards^(21)^. Some countries in Latin America may have recently gone through this transition, while others are still in the pre-transition stage. For example, a Chilean study using data from 2010 found that UPF consumption was highest in households in higher socio-economic strata (i.e. the pre-transition pattern)^(5)^, but a later study found that by 2015 the highest levels of UPF consumption were among household in the lowest strata (i.e. the post-transition pattern)^(15)^. Our findings highlight that labelling policies – which have a larger impact among those with higher levels of education – reduce differences between social strata in pre-transition contexts (because they reduce consumption in the higher-income groups who have the highest consumption) but widen differences in post-transition contexts^(15)^. A promising approach may be to combine tax and labelling policies, since taxes have a larger effect on households in the lower socio-economic strata and labelling has the greatest effect on the highest socio-economic strata.

As with any simulation study, a limitation is that the insights generated are tied to the model structure and parameters. The model is agnostic as to the specific mechanisms via which UPF prices, labelling and advertising affect UPF purchasing – rather, we identified effect estimates from relevant evaluation studies and the extant literature. Use of these effect estimates is both a strength and limitation of the study: it is a strength because we do not need to specify a specific causal structure via which policies achieve their effects, which may lessen the risk of bias from misspecification. However, an assumption inherent to this approach is that the effect sizes and elasticity estimates we used are valid and relevant to the Latin American context. Generally, studies of the own-price elasticity of multiple UPF products in multiple Latin American countries produced fairly similar estimates (i.e. –1 to –1·4)^(36–39)^.

Though few cities or countries have implemented mandatory UPF labelling laws, the recent evaluation study from Chile is an ideal model policy because it is likely similar to what would be passed in other Latin American countries and because the authors reported separate effect estimates by level of educational attainment. The stratified effects enabled us to assess how a labelling policy could reduce or exacerbate existing differences in UPF purchasing between social strata. Notably, the effect sizes are comparable to estimates from a meta-analysis of smaller-scale experimental studies conducted in the USA and Europe^(15)^. We were unable to identify a study of advertising effectiveness (i.e. the effect of exposure to advertising on food purchasing) in the Latin American context, either for food advertising or generally. We used an estimate of advertising elasticity from a meta-analysis of studies of advertising effectiveness^(41)^. The advertising elasticity implies a level of advertising ineffectiveness that our team found surprising given that companies spend billions of dollars per year on advertising. Nonetheless, the low advertising elasticity value is consistent with other values reported in the literature, including a recent study of advertising effects across a large number of products^(48)^. A caveat is that these studies were based on effect of television advertising, and point-of-purchase advertising, billboards, and other forms of advertising may be more effective. Given the pervasive level of UPF advertising in Latin American countries, an area for future research is evaluating the effect of different forms of UPF advertising on purchasing.

A further consideration is that the purpose of the model is to explore the effects of specific policy levers on UPF purchasing and does not include all drivers of UPF purchasing. Because we did not examine policies to change healthy food access, for example, the model is aspatial and does not consider agents’ proximity to healthy and unhealthy food retailers. Similarly, we did not consider the effects of product reformulation in response to taxes, labels and other policies^(49)^.

In this study, we presented a framework and virtual laboratory for exploring how available public policy levers can be used – both alone and in combination – to address high levels of UPF purchasing in Latin American countries at different stages of the social transition in UPF purchasing and with different UPF prices, purchasing levels, and social characteristics. Our results using UPF price, purchasing and social data from Mexico suggest that differential effectiveness of policies can either reduce or exacerbate differences in UPF purchasing between socio-economic strata. Given evidence suggesting that countries in Latin America are at different stages of the social transition in UPF purchasing, policymakers should consider the equity implications of policy as part of the planning process.

## Supplementary material

For supplementary material accompanying this paper visit https://doi.org/10.1017/S1368980021004833. click here to view supplementary material

## References

[1] Monteiro CA , Moubarac JC , Cannon G et al. (2013) Ultra-processed products are becoming dominant in the global food system. Obes Rev 14, Suppl. 2, 21–28. 24102801 10.1111/obr.12107

[2] Monteiro CA , Moubarac J-C , Levy RB et al. (2018) Household availability of ultra-processed foods and obesity in nineteen European countries. Public Health Nutr 21, 18–26. 28714422 10.1017/S1368980017001379 PMC10260838

[3] Marrón-Ponce JA , Sánchez-Pimienta TG , Louzada M et al. (2018) Energy contribution of NOVA food groups and sociodemographic determinants of ultra-processed food consumption in the Mexican population. Public Health Nutr 21, 87–93. 28937354 10.1017/S1368980017002129 PMC10260747

[4] Khandpur N , Cediel G , Obando DA et al. (2020) Sociodemographic factors associated with the consumption of ultra-processed foods in Colombia. Rev Saude Publica 54, 19. 32049210 10.11606/s1518-8787.2020054001176 PMC7006913

[5] Cediel G , Reyes M , da Costa Louzada ML et al. (2018) Ultra-processed foods and added sugars in the Chilean diet. Public Health Nutr 21, 125–133. 28625223 10.1017/S1368980017001161 PMC10260868

[6] Monteiro CA , Cannon G , Levy RB et al. (2019) Ultra-processed foods: what they are and how to identify them. Public Health Nutr 22, 936–941. 30744710 10.1017/S1368980018003762 PMC10260459

[7] Monteiro CA , Cannon G , Moubarac JC et al. (2018) The UN decade of nutrition, the NOVA food classification and the trouble with ultra-processing. Public Health Nutr 21, 5–17. 28322183 10.1017/S1368980017000234 PMC10261019

[8] Monteiro CA , Levy RB , Claro RM et al. (2010) Increasing consumption of ultra-processed foods and likely impact on human health: evidence from Brazil. Public Health Nutr 14, 5–13. 10.1017/S1368980010003241 21211100

[9] Marrón-Ponce JA , Flores M , Cediel G et al. (2019) Associations between consumption of ultra-processed foods and intake of nutrients related to chronic non-communicable diseases in Mexico. J Acad Nutr Diet 119, 1852–1865. 31262695 10.1016/j.jand.2019.04.020

[10] Costa CS , Del-Ponte B , Assunção MCF et al. (2018) Consumption of ultra-processed foods and body fat during childhood and adolescence: a systematic review. Public Health Nutr 21, 148–159. 28676132 10.1017/S1368980017001331 PMC10260745

[11] Canella DS , Levy RB , Martins APB et al. (2014) Ultra-processed food products and obesity in Brazilian households (2008–2009). PLoS One 9, e92752. 24667658 10.1371/journal.pone.0092752 PMC3965451

[12] Colchero MA , Rivera-Dommarco J , Popkin BM et al. (2017) In Mexico, evidence of sustained consumer response 2 years after implementing a sugar-sweetened beverage tax. Health Aff 36, 564–571. 10.1377/hlthaff.2016.1231 PMC5442881 28228484

[13] Taillie LS , Rivera JA , Popkin BM et al. (2017) Do high vs. low purchasers respond differently to a nonessential energy-dense food tax? Two-year evaluation of Mexico’s 8 % nonessential food tax. Prev Med 105, S37–S42. 10.1016/j.ypmed.2017.07.009 PMC5732875 28729195

[14] Caro JC , Ng SW , Taillie LS et al. (2017) Designing a tax to discourage unhealthy food and beverage purchases: the case of Chile. Food Policy 71, 86–100. 29375180 10.1016/j.foodpol.2017.08.001 PMC5783649

[15] Taillie LS , Reyes M , Colchero MA et al. (2020) An evaluation of Chile’s law of food labeling and advertising on sugar-sweetened beverage purchases from 2015 to 2017: a before-and-after study. PLoS Med 17, e1003015. 32045424 10.1371/journal.pmed.1003015 PMC7012389

[16] Reyes M , Garmendia ML , Olivares S et al. (2019) Development of the Chilean front-of-package food warning label. BMC Public Health 19, 906. 31286910 10.1186/s12889-019-7118-1 PMC6615240

[17] Soares Guimarães J , Mais LA , Marrocos Leite FH et al. (2020) Ultra-processed food and beverage advertising on Brazilian television by international network for food and obesity/non-communicable diseases research, monitoring and action support benchmark. Public Health Nutr 23, 2657–2662. 32468987 10.1017/S1368980020000518 PMC7477364

[18] Allemandi L , Castronuovo L , Tiscornia MV et al. (2018) Food advertising on Argentinean television: are ultra-processed foods in the lead? Public Health Nutr 21, 238–246. 28745262 10.1017/S1368980017001446 PMC10260822

[19] Correa T , Reyes M , Taillie LS et al. (2020) Food advertising on television before and after a national unhealthy food marketing regulation in Chile, 2016–2017. Am J Public Health 110, 1054–1059. 32437274 10.2105/AJPH.2020.305658 PMC7287518

[20] Colchero MA , Popkin BM , Rivera JA et al. (2016) Beverage purchases from stores in Mexico under the excise tax on sugar sweetened beverages: observational study. BMJ 352, h6704. 26738745 10.1136/bmj.h6704 PMC4986313

[21] Baker P , Machado P , Santos T et al. (2020) Ultra-processed foods and the nutrition transition: global, regional and national trends, food systems transformations and political economy drivers. Obes Rev 21, e13126. 32761763 10.1111/obr.13126

[22] Hammond RA , Ornstein JT , Fellows LK et al. (2012) A model of food reward learning with dynamic reward exposure. Front Comput Neurosci 6, 82. 23087640 10.3389/fncom.2012.00082 PMC3468814

[23] Christakis NA & Fowler JH (2007) The spread of obesity in a large social network over 32 years. N Engl J Med 357, 370–379. 17652652 10.1056/NEJMsa066082

[24] McPherson M , Smith-Lovin L & Cook JM (2001) Birds of a feather: homophily in social networks. Annu Rev Sociol 27, 415–444.

[25] Wright EO (1997) Class Counts: Comparative Studies in Class Analysis. Cambridge: Cambridge University Press.

[26] Marsden PV (1987) Core discussion networks of Americans. Am Sociol Rev 52, 122–131.

[27] Wilenski U (1999) NetLogo. Evanston, IL: Center for Connected Learning and Computer-Based Modeling, Northwestern University.

[28] Hall KD , Sacks G , Chandramohan D et al. (2011) Quantification of the effect of energy imbalance on bodyweight. Lancet 378, 826–837. 21872751 10.1016/S0140-6736(11)60812-X PMC3880593

[29] Confederação Nacional de Dirigentes Lojistas (2016) Facets of the Brazilian Woman: Consumer Market. São Paulo: SPC Brasil.

[30] Lelis CT , Teixeira KMD & Silva NMD (2012) Women’s inclusion in the workforce and its consequences for family dietary habits. Saúde Debate 36, 523–532.

[31] OECD (2019) Under Pressure: The Squeezed Middle Class. Paris: OECD Publishing.

[32] Marrón-Ponce J , Tolentino-Mayo L , Hernández-F M et al. (2019) Trends in ultra-processed food purchases from 1984 to 2016 in Mexican households. Nutrients 11, 45. 10.3390/nu11010045 PMC6356651 30587779

[33] Verbrugge LM (1983) A research note on adult friendship contact: a dyadic perspective. Soc F 62, 78.

[34] Hammond RA (2010) Social influence and obesity. Curr Opin Endocrinol Diabetes Obes 17, 467–471. 20689421 10.1097/MED.0b013e32833d4687

[35] Hammond RA & Ornstein JT (2014) A model of social influence on body mass index. Ann N Y Acad Sci 1331, 34–42. 24528150 10.1111/nyas.12344 PMC4133329

[36] Chacon V , Paraje G , Barnoya J et al. (2018) Own-price, cross-price, and expenditure elasticities on sugar-sweetened beverages in Guatemala. PLoS One 13, e0205931. 30346999 10.1371/journal.pone.0205931 PMC6197849

[37] Guerrero-López CM , Unar-Munguía M & Colchero MA (2017) Price elasticity of the demand for soft drinks, other sugar-sweetened beverages and energy dense food in Chile. BMC Public Health 17, 180. 28183287 10.1186/s12889-017-4098-x PMC5301435

[38] Paraje G (2016) The effect of price and socio-economic level on the consumption of sugar-sweetened beverages (SSB): the case of Ecuador. PLoS One 11, e0152260. 27028608 10.1371/journal.pone.0152260 PMC4814055

[39] Colchero MA , Salgado JC , Unar-Munguía M et al. (2015) Price elasticity of the demand for sugar sweetened beverages and soft drinks in Mexico. Econ Hum Biol 19, 129–137. 26386463 10.1016/j.ehb.2015.08.007

[40] Colchero MA , Guerrero-López CM , Molina M et al. (2016) Beverages sales in Mexico before and after implementation of a sugar sweetened beverage tax. PLoS One 11, e0163463. 27668875 10.1371/journal.pone.0163463 PMC5036812

[41] Hu Y , Lodish LM & Krieger AM (2007) An analysis of real world TV advertising tests: a 15-year update. J Advert Res 47, 341–353.

[42] Global Food Research Program (2021) Sugary Drink Taxes around the World. Chapel Hill, NC: University of North Carolina.

[43] Cawley J , Frisvold D , Hill A et al. (2019) The impact of the Philadelphia beverage tax on purchases and consumption by adults and children. J Health Econ 67, 102225. 31476602 10.1016/j.jhealeco.2019.102225

[44] Barquera S , Campos I & Rivera JA (2013) Mexico attempts to tackle obesity: the process, results, push backs and future challenges. Obes Rev 14, 69–78. 24103026 10.1111/obr.12096

[45] Backholer K , Blake M & Vandevijvere S (2017) Sugar-sweetened beverage taxation: an update on the year that was 2017. Public Health Nutr 20, 3219–3224. 29160766 10.1017/S1368980017003329 PMC10261626

[46] Basto-Abreu A , Barrientos-Gutiérrez T , Vidaña-Pérez D et al. (2019) Cost-effectiveness of the sugar-sweetened beverage excise tax in Mexico. Health Aff 38, 1824–1831. 10.1377/hlthaff.2018.05469 31682510

[47] Liu SY , Osgood N , Gao Q et al. (2016) Systems simulation model for assessing the sustainability and synergistic impacts of sugar-sweetened beverages tax and revenue recycling on childhood obesity prevention. J Oper Res Soc 67, 708–721.

[48] Shapiro B , Hitsch GJ & Tuchman A (2020) Generalizable and Robust TV Advertising Effects. NBER Working Paper. https://www.nber.org/papers/w27684 (accessed June 2020).

[49] Reyes M , Smith Taillie L , Popkin B et al. (2020) Changes in the amount of nutrient of packaged foods and beverages after the initial implementation of the Chilean law of food labelling and advertising: a non-experimental prospective study. PLoS Med 17, e1003220. 32722710 10.1371/journal.pmed.1003220 PMC7386631

[50] Chen Z (2019) An agent-based model for information diffusion over online social networks. Pap Appl Geogr 5, 77–97.

