# Supplementary material for: Potential impacts of policies to reduce purchasing of ultra-processed foods in Mexico at different stages of the social transition: an agent-based modelling approach
Source: Public Health Nutr. 2021 Dec 13;25(6):1711–9. doi: 10.1017/S1368980021004833 (PMC7612742; doi:10.1017/S1368980021004833)
Supplement: Supplementary file 1 [file S1368980021004833sup.zip › S1368980021004833sup001.docx]

**Description of SALURBAL food ABM based on PARTE framework**

**MODEL OVERVIEW**

The purpose of the ABM is to estimate the effects of tax and labeling policies on purchasing of ultra-processed food (UPF) in Latin American cities. We examine policy counterfactuals that include implementation of each policy separately and both policies combined. We also use the model to estimate the effects of the policies in scenarios in which the food industry responds to the policies by increasing advertising for UPF.

The model is coded in NetLogo.^1^

The model includes a population of 1,000 individual-agents with characteristics loosely based on adult females in **Mexico City, Mexico**. We focus on adult females because they are often the primary food purchasers in their households. The primary outcome is each agent’s weekly purchasing of ultra-processed food (UPF), which changes over time as the model runs. At each time step, which loosely represents one week, agents update their UPF purchasing based on the purchasing of their friends (social signal) and social norms. UPF purchasing is further updated based on changes in UPF prices, labeling and advertising. We use external data – typically from Mexico or Mexico City -- to inform baseline conditions in the model, including population income, educational attainment, and baseline UPF consumption. We chose this city because of data availability, and because Mexico City has implemented policies to reduce UPF consumption and policy evaluation data are available against which we can assess the model’s ability to reproduce observed trends as policies change.

**AGENT PROPERTIES**

The model includes one type of agent, representing adult female food consumers living in an urban area in Latin America. Individual agents are characterized by the following static state variables that are assigned at model initialization: income, education, and sensitivities to attitudes, price, and label information. Whenever possible, we used empirical sources to inform the distribution of these variables. We used data from the 2016 Mexican Survey of Household Income and Spending (ENIGH) to inform distributions of food purchasing, household income, and educational attainment. We restricted these analyses to households in the ENIGH sample with at least one adult female at least one child; although the model is intended to represent food purchasing in Latin American cities, we did not restrict the analyses to households in urban areas or households in a particular city. The variables, distribution parameters, and data sources are described in **Table A-1**. The values of the environmental parameters do not change as the model runs, but can be changed between scenarios (e.g., based on parameters from other countries or cities). UPF price and sensitivities to social influences (i.e., social influence and social norms), advertising, price, and labels are environment parameters that affect all agents in the simulation. The sensitivities quantify the effect of a one-unit change in a variable (i.e., price, label use or advertising) on a given agent's UPF purchasing. Other agent properties vary between agents and are drawn from distribution parameters described in the table below. These include parameters related to the distribution of UPF purchasing, income, education, and age.

**Table A-1. Variables and parameters that inform agent properties and actions**

| **Parameter** | **Symbol** | **Variable Type & Unit** | **Initialization** | **Update** | **Notes** |
| --- | --- | --- | --- | --- | --- |
| UPF purchasing | ${UPF}_{i,t}$ | Continuous. kCal purchased per week. | Drawn from Poisson distribution. In the pre-transition scenario, mean = 3446 for high-income agents, and mean = 2966 for low-income agents. In the post-transition scenario, mean = 2620 for high-income agents, and mean = 3100 for low-income agents. | Yes | Agent property. Baseline UPF purchasing for each agent is drawn from two separate distributions for high- and low-income agents. The means of these two distributions was determined by calibrating the overall simulated mean UPF purchasing at the population level against average purchasing reported by Marron-Ponce et al. (2019).^2^ They report total daily consumption of 1875.4 kCal/day/adult equivalent among Mexican households, with UPF consumption being 23.1% of total consumption. Thus, UPF purchasing is 3,033 kCal/wk (1875.4*7*23.1%). |
| UPF price. | $p$ | Continuous. Mexican pesos (MXN) | Global variable which is adjustable by slider. The price paid for the average quantity of UPF purchased per week for a family of four is set to 193 MXN in the baseline model. | No | The price of UPF factors in only in the context of the tax policy where it is used to calculate the relative change in cost and its impact on household UPF purchasing. Based on Mexico City data derived from Table 2 in Marron-Ponce et al. (2019). They report that total food spending is 27.3 MXN per person per day and that 25.2% of spending is on UPF. Thus, spending is 193 MXN per family per week (i.e., 27.3*7 days*25.2%*4 people in the household). |
| Education |  | Categorical. | Randomly assigned such that 27% of agents have  ≥high school education and the rest (73%) have <high school education. | No | Agent property. Based on data from the ENIGH 2016 survey.^3^ |
| Income | ${inc}_{i}$ | Continuous | Randomly drawn from separate log-normal distributions for low-education agents (Mean: 888.79, SD: 911.21) and high-education agents (Mean: 2043.54, SD: 2224.77). | No | Agent property. Unit of income is pesos per week. Informed by 2016 ENIGH data for households with >= 1 child and >= 1 woman (median household size = 4 people).^3^ |
| Income group |  | Categorical. Two groups: 1) high income, 2) low-middle income. | High income status was defined as households with > 1890 pesos/wk (which is 7,561 pesos/ month). |  | Agent property. Income group is based on continuous income (above) and used to construct each agent’s social network and social comparison group, as described below. High-income threshold based on OECD estimates for a 4-person household.^4^ Median household size of 4 for households with a child is from the 2016 ENIGH data.^3^ |
| Age |  | Categorical. with 3 age categories: young, middle-aged, and older adult. | Randomly drawn such that 25% of agents are the young and the older age bands, and 50% are in the middle-aged band. | No | Agent property. Randomly drawn from uniform distribution. Same across scenarios. |
| Resistance to change in UPF based on social influences (social signal & social norms) | $e_{r}$ | Continuous. $e_{r}=10$ | Calibrated parameters; | No | Environment parameter. Resistance to social signal and social norms associated with UPF purchasing are set to be equivalent based on lack of empirical evidence in support of different values. Calibrated to reproduce UPF purchasing observed in the 2016 ENIGH data. The simulation outcome was the average over 208 time steps (excluding the 100 time step burn-in period). |
| Sensitivity to advertising | $e_{adv}$ | Continuous. | Based on advertising elasticity value = 0.113 (i.e., a 1% increase in UPF advertising is associated with a 0.113% increase in purchasing) | No | Environment parameter. Informed by Hu, Lodish, and Krieger (2007).^5^ |
| Sensitivity to price | $e_{p}$ | Continuous. | Based on own-price elasticity of sugar-sweetened beverage =  -1.2 (i.e., a 1% increase in the price of UPF is associated with a 1.2% decline in purchasing). | No | Implemented as an environment parameter but applied to the relative price of UPF (i.e., UPF price expressed as a percentage of agents’ income) rather than the absolute cost to account for heterogeneity in agents’ price sensitivity.  Value informed by studies of price elasticities of SSB in several Latin American countries, all of which range from -1 to -1.4.^6-9^ |
| Sensitivity to labeling | $e_{l,i}$ | Continuous. | Effect size varies by education-status: low education effect size = -0.22 & high education effect size = -0.29. | No | Environment parameter based on evaluation study from nutrition warning label policy in Chile by Taillie et al (2020).^10^ |

There is one set of global variables that updates as the model runs: the mean weekly UPF purchasing for all agents in each sub-group. Sub-groups are described below but are defined by income group (high vs. low), education (high vs. low), and age (younger, middle aged, older). The global variable is in **Table A-2**.

**Table A-2. Global variables**

| **Variable** | **Type** | **Update** | **Notes** |
| --- | --- | --- | --- |
| UPF purchasing norms based on age, gender, education | Double. Mean UPF purchasing for each subgroup. | Updates each time step after all agent actions | Initially calculated at time step 0 after initialization of population and environment. |

**ACTIONS**

**Overview**

Agents engage in the following actions: 1) construct a group of other agents that will exert a social signal regarding UPF purchasing (e.g., friends, family), 2) calculate average UPF purchasing among all members of the friendship network (social signal), 3) calculate average UPF purchasing among the population of agents with the same demographic characteristics (social norms), 4) update UPF purchasing based on social influences, 5) update UPF purchasing further based on any changes in UPF price, labels or advertising campaigns. Within each module, agents are processed in sequential order. If none of the policies are active, UPF purchasing will only be updated based on social influences. This is because the baseline UPF purchasing assigned to agents already considers the price of UPF.

**Social influence adjustments (i.e., social signal and social norms)**

*Construct the social signal group for each agent*

This module is run once per replication, at time step 0 after the environment and population have been initialized. Every agent constructs a sample of at least 3 and a maximum of 50 agents with similar social characteristics. There were few available studies to inform the degree and other characteristics of the social network – in particular, each alter in an agent’s social network represents a female in a household with children that influences (e.g., via discussion, observation, etc) the agent’s food purchasing behavior. The agent constructs the social network by selecting one agent at a time from the population and adding that agent to the sample. The probability that a given agent will be selected is proportionate to the social similarity score between that agent and the agent doing the selecting. Social similarity scores are assessed using the scoring in **Table A-3**, in which agent characteristics are given unitless points.

**Table A-3. Social similarity score**

| **Characteristic & Level** | **Points** |
| --- | --- |
| *Income (lower vs. higher)* |  |
| Same income group | 1 |
| Different income group | 0 |
| *Education (lower vs. higher)* |  |
| Same education | 1 |
| Different education | 0 |
| *Age (younger, middle aged, older)* |  |
| Same age group | 1 |
| Off by one level (e.g.,middle vs. older) | 0.5 |
| Off by two levels (i.e., younger vs. older) | 0 |

The diversity of the friendship networks created can be adjusted using the “scaleVar” slider in the model, where higher values increase the similarity of agents in the friendship network (i.e., the higher the value the less demographic diversity there is on average in the friendship networks. The model uses a value of scaleVar = 2; this value was determined through calibration of the parameter to reproduce UPF purchasing patterns in the 2016 ENIGH data.

The characteristics of the small-world network created in the ABM are featured in **Table A-4** and compared to those of an artificial small world network. We used a command that uses the “nw” extension in NetLogo to calculate the characteristics of the social network. A key difference between the two networks is that the food ABM network has a higher local clustering coefficient than a typical small world network. This is an artifact of the similarity scores on which connections between agents were conditioned. That is, we wanted to have more clustering among people that were similar, above and beyond what we might ordinarily see in a small world network.

**Table A-4. Network characteristics**

| Network | N | Number of links | Proportion of links / nodes | Average degree | Mean path length | Local clustering coefficient | Global clustering coefficient |
| --- | --- | --- | --- | --- | --- | --- | --- |
| Typical small world | 800 | 2337 | 2.921 | 5.842 | 4.3068 | 0.007 |  |
| ABM network | 1000 | 2744 | 2.744 | 5.47 | 4.24 | 0.0127 | 0.0023 |

Note: characteristics of the typical network are from Chen (2019)^11^

*Update UPF purchasing*

To update purchasing, each agent performs a series of "adjustments." The first adjustments reflect each agent’s desire to conform to within a threshold distance of the UPF purchasing among two groups: 1) agents in their social network (i.e., their friends), and 2) the population of agents with similar characteristics. For the social signal (i.e., friends) adjustment, agent i compares its food purchasing level to the average level among its friends. If agent $i$’s purchasing is more than a threshold distance -- 50kcal/wk -- from the average of her set of friends $f$, she shifts her purchasing to be a small amount closer to the norm. The threshold cutoff represents the balance between individuals’ tolerance for individuality versus their desire to constrain to the social signal and social norms, as well as uncertainty in people’s knowledge of the true levels of UPF purchasing of their friends. The magnitude of the shift is the difference between agent $i$’s UPF purchasing and the average purchasing of her friends, multiplied by a factor of ${1/e}_{r}$ – this represents agents’ resistance to social influences.

$$if \left\{ \left| \bar{{UPF}_{f,t-1}}-{UPF}_{i, t-1} \right|>50 \right\}$$

$$then \left\{ {UPF}_{i,t}= {UPF}_{i,t-1}+ \frac{1}{e_{r}=10}({\bar{{UPF}_{f,t-1}}-UPF}_{i,t-1}) \right\}$$

The same process is repeated for social norms, but the comparator is the average purchasing level of the set of agents $n$ that share the same age group (i.e., younger, middle aged, older), income (i.e., lower, higher) and education status (i.e., lower, higher) as agent $i$.

**Policies and Environmental Changes**

UPF purchasing adjustments described below occur in response to the implementation of three different policies: 1) UPF taxes, 2) UPF labels, 3) changes in UPF advertising levels. These adjustments occur only once at the time the policy is implemented and are thereafter assumed to be active and embodied within the UPF purchasing patterns of the population for all subsequent time steps.

*UPF Tax*

Each agent’s UPF purchasing is updated following implementation of the UPF tax, which increases the price of UPF. The update magnitude is based on the magnitude of the increase in the UPF price and the price elasticity $e_{p}$ of UPF. We used studies from Chile, Ecuador, and the US to identify the price elasticity of UPF; estimates ranged from -1 to -1.4. We used the midpoint of this range (i.e., -1.2). This means that a 1% increase in the weekly price of UPF is associated with a 1.2% decrease in weekly UPF purchasing in the model.

The standard price elasticity equation is given by:

$$e_{p}=\frac{\% change in UPF}{\% change in price}=\frac{{(UPF}_{i,t\left( 2 \right)}-{UPF}_{i,t\left( 1 \right)})/{UPF}_{i,t\left( 1 \right)}}{\% change in price}$$

This equation can be re-arranged to the following:

$${UPF}_{i,t(2)}= {UPF}_{i,t(1)}+price elasticity*change in price* {UPF}_{i,t(1)}$$

Due to the high level of income inequality in Latin American cities, we implemented the price elasticity based on the relative price of UPF. The reason is that, on a relative basis, UPF is much more expensive among those in lower income strata and, as a result, taxes are likely to have a larger effect among these groups. Each agent’s UPF purchasing is updated as follows:

|  | ${UPF}_{i,t(2)}= {UPF}_{i,t(1)}+e_{p}*\left( \frac{rp_{n,i}-rp_{b,i}}{rp_{b,i}} \right)* {UPF}_{i,t(1)}$ | (2) |
| --- | --- | --- |

Where, the relative price at baseline $rp_{b,i}$ and relative new price $rp_{n,i}$of weekly UPF, respectively, are calculated by dividing the weekly baseline or new price of UPF by agent $i$’s weekly income ${inc}_{i}$:

$rp_{b,i}= \frac{p_{b,i}}{{inc}_{i}}$ and $rp_{n,i}= \frac{p_{n,i}}{{inc}_{i}}$

*UPF Labeling Policy*

UPF purchasing is further adjusted when a labeling policy is implemented. The label sensitivity estimates in the model were informed by an evaluation of front-of-package labeling policy on high-in-calorie beverage purchasing in Chile conducted by Taillie et al (2020).^10^ We used separate effect estimates for those with high educational attainment (29% reduction) and low educational attainment (22% reduction). After the labeling policy is implemented, each agent’s UPF purchasing level is updated as follows:

|  | ${UPF}_{i,t(2)}= {UPF}_{i,t(1)}*(1+e_{l,i})$ | (4) |
| --- | --- | --- |

*Increase in UPF Advertising (industry response)*

A similar adjustment in UPF purchasing occurs if there is a change in the level of advertising $\Delta adv$ from baseline. We examine both increases and decreases in advertising. Increases reflect efforts from the food industry to offset the effects of UPF reduction policies by increasing advertising. Decreases reflect policies to restrict advertising levels. We used an advertising elasticity estimate from Hu, Lodish, and Krieger (2007), who found that a 1% increase in advertising was associated with a 0.113% increase in UPF purchasing.^5^ Each agent’s UPF purchasing is updated based on the magnitude of the change in advertising levels and the advertising elasticity $e_{adv}$, as follows:

$${UPF}_{i,t(2)}= {UPF}_{i,t(1)}+advertising elasticity*change in advertising* {UPF}_{i,t(1)}$$

|  | ${UPF}_{i,t(2)}= {UPF}_{i,t(1)}+e_{adv}*\Delta adv* {UPF}_{i,t(1)}$ | (3) |
| --- | --- | --- |

**Table A-5. Main data sources for parameters to be used for sensitivity weights**

| **Description** | **Value** | **Source & Notes** |
| --- | --- | --- |
| Price elasticity of UPF | -1.2 (i.e., a 1% increase in the price of UPF is associated with a 1.2% decline in purchasing). | In range from own-price elasticities of SSB from Chile, Ecuador, Mexico and elsewhere.^7,8,12^ Generally, studies observe elasticities in the -1 to -1.4 range. |
| Effect of front-of-package labeling policy | Reduction in UPF consumption of 22% among low-education households and 29% among high-education households. | Taillie et al (2020) evaluation of front-of-package labeling policy on high-in-calorie beverage purchasing in Chile: Compared to the counterfactual, the volume of high-in beverage purchases decreased 22.8 mL/capita/day, post-regulation (95% confidence interval [CI] −22.9 to −22.7; p < 0.001), or 23.7% (95% CI −23.8% to −23.7%).^10^ Effects were -22% among low-education households and 29% among high-education households. |
| Advertising elasticities | Average elasticity of weight tests = 0.113 (SD of elasticity within each test = 0.139). Standardized mean difference in Ad/NoAd tests = 2.7 (Mean adjusted sales volume post – pre)/SD. | Hu, Lodish, and Krieger (2007) – follow-up on seemingly famous study in marketing. Analysis of MarketScan tests of weight and copy.^5^ General design: MarketScan participants in a given market are exposed to an experimental condition and their purchasing is compared to that of participants in a matched comparison market. |

**TIME**

Each time step of the model represents one week. Each simulation is run for 308 time steps, which includes a 100 time step burn-in period required for the model to reach equilibrium. The remaining 208 time steps, representing roughly a period of 4 years will form the focus of the present paper and the policy scenarios discussed. Policies are implemented after the 52^nd^ time step, not including the burn-in periods.

**ENVIRONMENT**

The model is not spatially explicit, meaning that there is no agent movement and the physical environment plays no role in the function of the model. The agent population size is set at 1,000 agents.

**POLICIES**

We run the model under the following simulation scenarios meant to represent policies to reduce UPF purchasing:

1. Baseline:
   - The UPF price is based on UPF spending data from Mexico City, as described in Table A-1. No mandatory UPF labeling. Advertising set at a baseline value (1.0, which is arbitrary).
2. Policies Alone
   - Mandatory UPF labeling
   - UPF Taxes
     1. 8% (actual junk food in Mexico)
     2. 10% (actual SSB tax in Mexico)
     3. 20% (considered SSB tax in Mexico)
     4. 50% (very large)
   - Advertising
     1. +25%
     2. +50%
     3. -25%
     4. -50%
3. Policy combinations
   - 50% tax and label, 50% decrease in advertising (policy combination at maximum levels)
   - 50% tax and label, no advertising change (no industry response)
   - 50% tax and label, 50% increase in advertising (industry response to maximum tax and labeling)
   - 8% tax and label, 25% decrease in advertising (minimum combination)
   - 8% tax and label, no advertising change (no industry response)
   - 8% tax and label, 25% increase in advertising (industry response to minimum tax and labeling)

**CALIBRATED PARAMETERS**

We used model calibration to set the values of four unknown parameters in the model: resistance to conforming to social signal and social norms ($e_{r}$), the effect of the social similarity score in generating the social network (scaleVar, which at higher levels leads to more homophily in social networks), the mean UPF purchasing of low-income agents at baseline, and the mean UPF purchasing of high-income agents at baseline. We used the following calibration criteria:

1. The average, equilibrium state weekly household UPF purchasing at the population-level must be within 5 kCal of the calibration target of 3033 kCal per week (informed by the Mexico study).
2. The calibrated model parameters must maintain unique distributions of weekly household UPF purchasing by income.
3. The selected model configuration will be one that meets both calibration criteria 1 and 2.

We ran the model for 308 time steps (or 308 weeks), and 400 repeated simulations. Given that the UPF purchasing stabilized after 100 time steps (i.e., reaches equilibrium), only the last 208 time steps were analyzed and compared to the above criteria. The below parameter configuration was found to meet the calibration criteria.

All Scenarios:

Resistance to conforming to social signal and norms $\boldsymbol{(}e_{r})$ = 10

Diversity in the friendship network (scaleVar) = 2

Pre-Social Transition Scenarios:

Mean UPF purchasing of low-income agents = 2966

Mean UPF purchasing of upper-middle-income agents =3446

Post-Social Transition Scenarios:

Mean UPF purchasing of low-income agents = 3100

Mean UPF purchasing of upper-middle-income agents =2620

**Figure 1-A** shows the fit between the simulated model output (blue line with 95%CI bands) and the calibration target (red dashed line) informed by Marron-Ponce et al. (2019), a study of UPF purchasing in Mexico.^2^ The blue line represents the averaged effect of 400 repeated simulations for each of the 208 time steps.


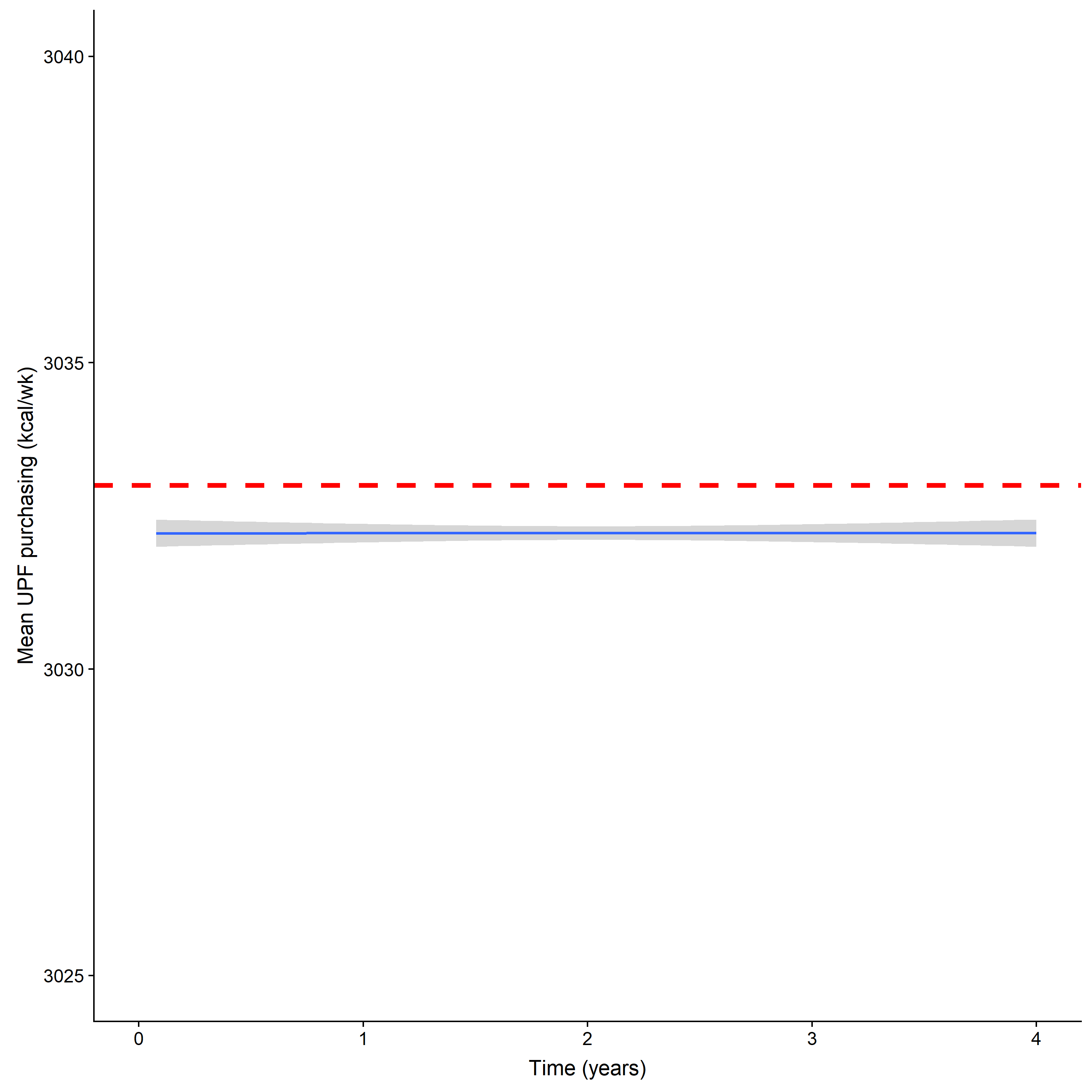


**Figure 1-A**

To determine the optimal number of runs for each policy scenario, we calculated the average weekly UPF purchasing (with 95%CIs) for different numbers of repeated runs ranging from 50 to 400 (**Figure 2-A**). We ultimately decided to simulate policy scenarios using 200 repeated simulations as only relatively small variations in weekly UPF purchasing were observed for simulations with 200 runs or more.


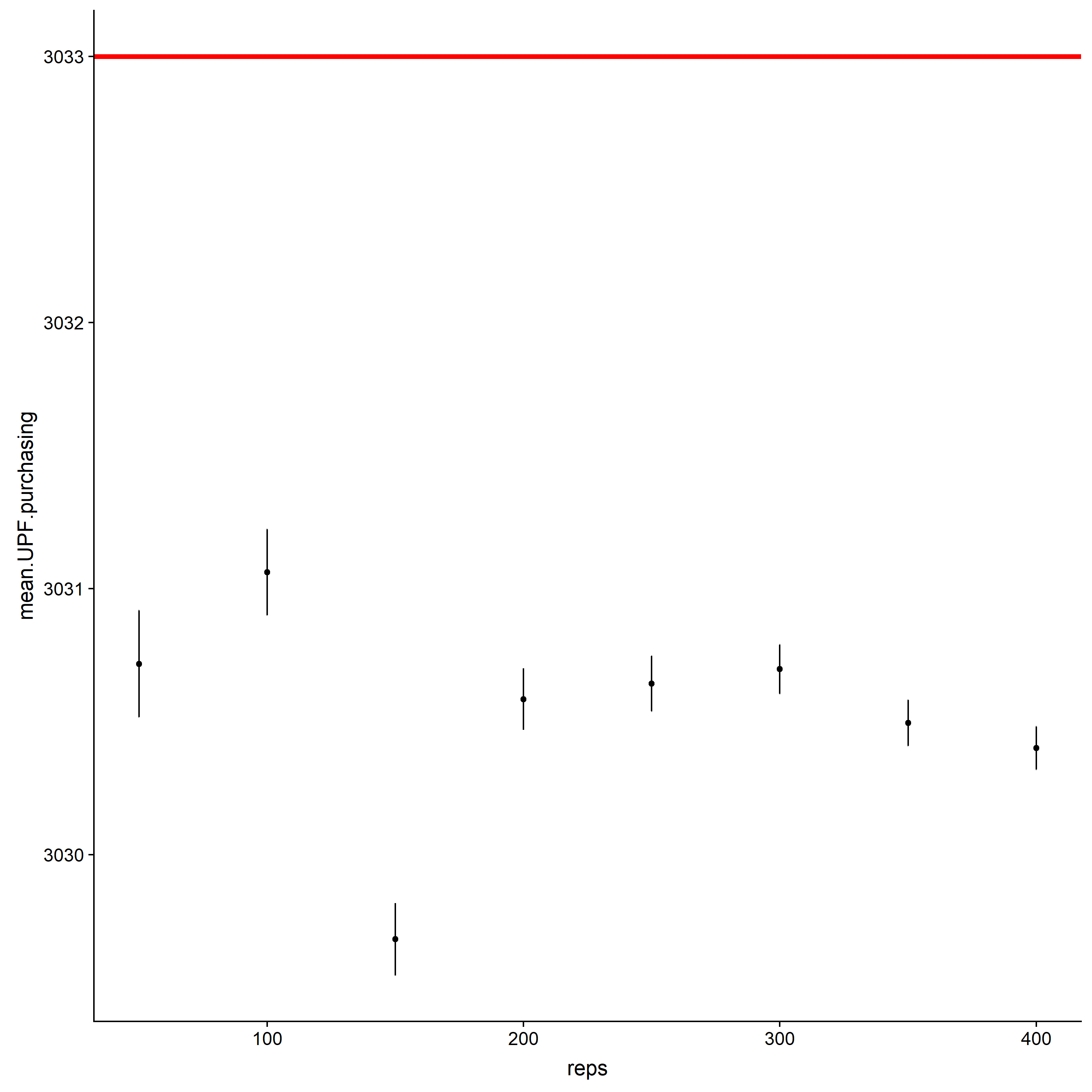


**Figure 2-A**

**Bibliography**

1. Wilenski U. NetLogo. In: Evanston, IL: Center for Connected Learning and Computer-Based Modeling, Northwestern University; 1999: <http://ccl.northwestern.edu/netlogo/>.

2. Marrón-Ponce J, Tolentino-Mayo L, Hernández-F M, Batis C. Trends in ultra-processed food purchases from 1984 to 2016 in Mexican households. *Nutrients.* 2019;11(1):45.

3. Instituto Nacional de Estadística GeII. Encuesta Nacional de Ingresos y Gastos de los Hogares 2016 (National Survey of Household Income and Spending). In: INEGI, ed. Aguascalientes, Aguascalientes, Mexico: INEGI; 2016: <https://www.inegi.org.mx/programas/enigh/nc/2016/>.

4. OECD. *Under Pressure: The Squeezed Middle Class.* 2019.

5. Hu Y, Lodish LM, Krieger AM. An analysis of real world TV advertising tests: A 15-year update. *Journal of Advertising Research.* 2007;47(3):341-353.

6. Chacon V, Paraje G, Barnoya J, Chaloupka FJ. Own-price, cross-price, and expenditure elasticities on sugar-sweetened beverages in Guatemala. *PLOS ONE.* 2018;13(10):e0205931.

7. Guerrero-López CM, Unar-Munguía M, Colchero MA. Price elasticity of the demand for soft drinks, other sugar-sweetened beverages and energy dense food in Chile. *BMC public health.* 2017;17(1):180.

8. Paraje G. The effect of price and socio-economic level on the consumption of sugar-sweetened beverages (SSB): the case of Ecuador. *PLoS One.* 2016;11(3):e0152260.

9. Colchero MA, Salgado JC, Unar-Munguía M, Hernández-Ávila M, Rivera-Dommarco JA. Price elasticity of the demand for sugar sweetened beverages and soft drinks in Mexico. *Economics & Human Biology.* 2015;19:129-137.

10. Taillie LS, Reyes M, Colchero MA, Popkin B, Corvalán C. An evaluation of Chile’s Law of Food Labeling and Advertising on sugar-sweetened beverage purchases from 2015 to 2017: A before-and-after study. *PLOS Medicine.* 2020;17(2):e1003015.

11. Chen Z. An Agent-Based Model for Information Diffusion over Online Social Networks. *Papers in Applied Geography.* 2019;5(1-2):77-97.

12. Batis C, Rivera JA, Popkin BM, Taillie LS. First-year evaluation of Mexico’s tax on nonessential energy-dense foods: an observational study. *PLoS medicine.* 2016;13(7):e1002057.
