# Supplementary material for: Potential impacts of policies to reduce purchasing of ultra-processed foods in Mexico at different stages of the social transition: an agent-based modelling approach
Source: Public Health Nutr. 2021 Dec 13;25(6):1711–9. doi: 10.1017/S1368980021004833 (PMC7612742; doi:10.1017/S1368980021004833)
Supplement: Supplementary file 1 [file S1368980021004833sup.zip › S1368980021004833sup002.docx]

| **Supplemental Results Table 1: Mean weekly purchasing of ultra-processed foods in kCal/week by educational attainment strata, in a population in which UPF purchasing is greater in households with higher income (pre-social transition)** | | | | | | | |
| --- | --- | --- | --- | --- | --- | --- | --- |
|  | **Higher Education** | | **Lower Education** | | **Difference (Lower - Higher)** | | |
| **Policy** | **Step 208 Mean (SD)** | **Step 52 Mean (SD)** | **Step 208 Mean (SD)** | **Step 208 Mean (SD)** | **At Step 52** | **At Step 208** | **Relative Change in Difference** |
| Tax = 0%, Label = Off, Advertising = 0% | 3064 (17) | 3064 (17) | 3015 (13) | 3015 (13) | -50 | -50 | 0% |
| Tax = 0%, Label = Off, Advertising = 25% | 3064 (17) | 3149 (17) | 3015 (13) | 3099 (13) | -50 | -50 | 0% |
| Tax = 0%, Label = Off, Advertising = 50% | 3064 (17) | 3234 (17) | 3015 (13) | 3184 (13) | -50 | -50 | 0% |
| Tax = 0%, Label = On, Advertising = 0% | 3064 (17) | 2227 (14) | 3015 (13) | 2325 (10) | -50 | 98 | -297% |
| Tax = 8%, Label = Off, Advertising = 0% | 3064 (17) | 2771 (15) | 3015 (13) | 2726 (11) | -50 | -45 | -10% |
| Tax = 10%, Label = Off, Advertising = 0% | 3064 (17) | 2697 (15) | 3015 (13) | 2653 (11) | -50 | -44 | -12% |
| Tax = 20%, Label = Off, Advertising = 0% | 3064 (17) | 2329 (13) | 3015 (13) | 2291 (10) | -50 | -38 | -24% |
| Tax = 50%, Label = Off, Advertising = 0% | 3064 (17) | 1226 (7) | 3015 (13) | 1206 (5) | -50 | -20 | -60% |
| Tax = 50%, Label = On, Advertising = -50% | 3064 (17) | 826 (5) | 3015 (13) | 885 (4) | -50 | 59 | -218% |
| Tax = 50%, Label = On, Advertising = 0% | 3064 (17) | 877 (5) | 3015 (13) | 938 (4) | -50 | 61 | -222% |
| Tax = 50%, Label = On, Advertising = 50% | 3064 (17) | 927 (5) | 3015 (13) | 990 (4) | -50 | 63 | -227% |
| Tax = 8%, Label = On, Advertising = -25% | 3064 (17) | 1952 (12) | 3015 (13) | 2046 (9) | -50 | 93 | -288% |
| Tax = 8%, Label = On, Advertising = 0% | 3064 (17) | 2010 (13) | 3015 (13) | 2105 (9) | -50 | 94 | -290% |
| Tax = 8%, Label = On, Advertising = 25% | 3064 (17) | 2068 (13) | 3015 (13) | 2163 (9) | -50 | 95 | -292% |

| **Supplemental Results Table 2: Mean weekly purchasing of ultra-processed foods in kCal/week by educational attainment strata, in a population in which UPF purchasing is greater in households with lower income (post-social transition)** | | | | | | | |
| --- | --- | --- | --- | --- | --- | --- | --- |
|  | **Higher Education** | | **Lower Education** | | **Difference (Lower - Higher)** | | |
| **Policy** | **Step 52 Mean (SD)** | **Step 208 Mean (SD)** | **Step 52 Mean (SD)** | **Step 208 Mean (SD)** | **At Step 52** | **At Step 208** | **Relative Change in Difference** |
| Tax = 0%, Label = Off, Advertising = 0% | 3002 (16) | 3002 (16) | 3050 (13) | 3050 (13) | 48 | 48 | 0% |
| Tax = 0%, Label = Off, Advertising = 25% | 3002 (16) | 3088 (16) | 3050 (13) | 3137 (13) | 48 | 48 | 0% |
| Tax = 0%, Label = Off, Advertising = 50% | 3002 (16) | 3175 (16) | 3050 (13) | 3223 (13) | 48 | 48 | 0% |
| Tax = 0%, Label = On, Advertising = 0% | 3002 (16) | 2208 (19) | 3050 (13) | 2355 (14) | 48 | 147 | 204% |
| Tax = 8%, Label = Off, Advertising = 0% | 3002 (16) | 2714 (14) | 3050 (13) | 2758 (11) | 48 | 44 | -10% |
| Tax = 10%, Label = Off, Advertising = 0% | 3002 (16) | 2642 (14) | 3050 (13) | 2684 (11) | 48 | 42 | -12% |
| Tax = 20%, Label = Off, Advertising = 0% | 3002 (16) | 2281 (12) | 3050 (13) | 2318 (10) | 48 | 37 | -24% |
| Tax = 50%, Label = Off, Advertising = 0% | 3002 (16) | 1201 (6) | 3050 (13) | 1220 (5) | 48 | 19 | -60% |
| Tax = 50%, Label = On, Advertising = -50% | 3002 (16) | 811 (5) | 3050 (13) | 895 (4) | 48 | 84 | 74% |
| Tax = 50%, Label = On, Advertising = 0% | 3002 (16) | 861 (5) | 3050 (13) | 948 (4) | 48 | 87 | 81% |
| Tax = 50%, Label = On, Advertising = 50% | 3002 (16) | 911 (6) | 3050 (13) | 1002 (5) | 48 | 91 | 89% |
| Tax = 8%, Label = On, Advertising = -25% | 3002 (16) | 1931 (16) | 3050 (13) | 2072 (11) | 48 | 141 | 192% |
| Tax = 8%, Label = On, Advertising = 0% | 3002 (16) | 1989 (17) | 3050 (13) | 2131 (12) | 48 | 142 | 195% |
| Tax = 8%, Label = On, Advertising = 25% | 3002 (16) | 2047 (17) | 3050 (13) | 2191 (12) | 48 | 143 | 197% |
